# Phase and composition controllable synthesis of cobalt manganese spinel nanoparticles towards efficient oxygen electrocatalysis

**DOI:** 10.1038/ncomms8345

**Published:** 2015-06-04

**Authors:** Chun Li, Xiaopeng Han, Fangyi Cheng, Yuxiang Hu, Chengcheng Chen, Jun Chen

**Affiliations:** 1 Key Laboratory of Advanced Energy Materials Chemistry (Ministry of Education) and State Key Laboratory of Elemento-Organic Chemistry, Nankai University, Tianjin 300071, China; 2 Collaborative Innovation Center of Chemical Science and Engineering (Tianjin), Nankai University, Tianjin 300071, China

## Abstract

Spinel-type oxides are technologically important in many fields, including electronics, magnetism, catalysis and electrochemical energy storage and conversion. Typically, these materials are prepared by conventional ceramic routes that are energy consuming and offer limited control over shape and size. Moreover, for mixed-metal oxide spinels (for example, Co_*x*_Mn_3−*x*_O_4_), the crystallographic phase sensitively correlates with the metal ratio, posing great challenges to synthesize active product with simultaneously tuned phase and composition. Here we report a general synthesis of ultrasmall cobalt manganese spinels with tailored structural symmetry and composition through facile solution-based oxidation–precipitation and insertion–crystallization process at modest condition. As an example application, the nanocrystalline spinels catalyse the oxygen reduction/evolution reactions, showing phase and composition co-dependent performance. Furthermore, the mild synthetic strategy allows the formation of homogeneous and strongly coupled spinel/carbon nanocomposites, which exhibit comparable activity but superior durability to Pt/C and serve as efficient catalysts to build rechargeable Zn–air and Li–air batteries.

## Abstract (web summary)

It is challenging to synthesize cobalt manganese spinels with controlled phase and composition. Here the authors present a solution-based synthesis method for the spinels, which show potential in catalysing oxygen reduction reactions.

## Graphical abstract

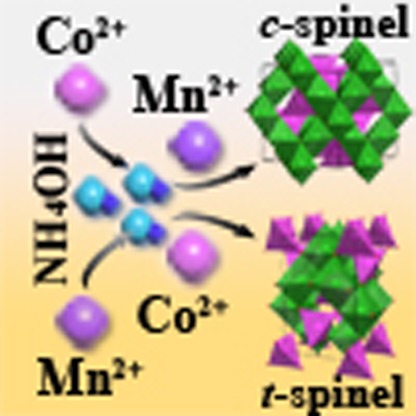

Spinel-type compounds with the general formula of AB_2_X_4_ (A, B=metal, X=chalcogen) have attracted extensive interest because of their diverse properties and wide applications in electronics, catalysis, magnetism and electrochemical technologies such as batteries, fuel cells and electrolysers^1^,^2^,^3^,^4^. Spinels are traditionally synthesized through solid-state methods involving grinding and firing the mixtures of the corresponding metal oxides, nitrates or carbonates^5^,^6^, which require elevated temperature and prolonged time to overcome the reaction energy barriers^7^. The prepared spinels often show irregular shape, large particle size and low surface area, seriously affecting their physicochemical properties. Continuous efforts have been dedicated to developing alternative mild synthetic strategies such as hydro/solvothermal route^8^, sol–gel method^9^ and carbonate co-precipitation^10^. However, it remains challenging to prepare homogenous and ultrasmall nanocrystalline spinels under mild conditions.

As a typical mixed transition metal spinel, cobalt manganese oxides (Co_3−*x*_Mn_*x*_O_4±δ_) have versatile applications as lithium storage electrode, catalysts and magnetic materials^8^,^9^,^10^,^11^,^12^. The rational preparation of cobalt manganese spinels is made complex by different structural variations multiple oxidation states and elusive distribution of cations. Moreover, the crystallographic phases of Co_3−*x*_Mn_*x*_O_4_ are sensitively determined by the Co/Mn ratio, namely, high Mn content (1.9<*x*≤3) tends to form the tetragonal phase while low proportions of Mn (0≤*x*≤1.3) result in the cubic spinel at room temperature^8^,^11^. The Mn-enriched cubic spinel (*x*=1.8) is observed at temperature around 998 K but is not stable at low temperature and immediately transforms into the tetragonal structure when quenching^13^. There seems to be a compositional limit to obtaining single cubic or tetragonal spinel phase at mild conditions. Theoretically, the lattice distortion caused by Jahn–Teller effect of Mn^3+^ (3*d*^4^) in octahedral interstices is responsible for the lowering of crystallographic symmetry from cubic to tetragonal phases^14^,^15^. If the cation Mn^3+^ concentration is low enough (≤60–65%), Jahn–Teller distortion would not be prominent and the spinels could exist in cubic form^16^. Therefore, it is feasible but remains a great challenge to rationally synthesize Co–Mn spinels with controlled phase over a wide composition range, which is highly desired in structure-sensitive applications.

The catalytic oxygen reduction/evolution reactions (ORR/OER) determine performance of different electrochemical devices, including fuel cells, metal–air batteries and direct solar or electricity-driven water splitting^17^,^18^,^19^. Noble metal and alloys are the most active oxygen electrocatalysts^20^, but are expensive and scarce. Substantial efforts have been dedicated to improving the performance of Pt-based catalysts with minimal Pt loading^21^,^22^,^23^,^24^ or developing alternative catalysts based on non-noble elements^25^,^26^,^27^,^28^,^29^,^30^. Multivalent metal spinels, Co_3−*x*_Mn_*x*_O_4±δ_ in particular, are considered as attractive non-precious oxygen electrocatalysts^8^,^11^,^29^. However, the structure–properties relationship of spinel metal oxides remains elusive and their catalytic performances are limited relative to their Pt-based counterparts, because of low electrical conductivity and poor O_2_-binding and -activating ability.

To develop efficient mixed 3*d*–metal–spinel catalysts with the above factors in mind, we describe herein a unique strategy for the rational synthesis of nanocrystalline Co_3−*x*_Mn_*x*_O_4_ (1≤*x*≤2) as well as NiCo_2_O_4_, FeCo_2_O_4_, ZnCo_2_O_4_ and ZnMn_2_O_4_. This solution-based synthesis involves oxidizing precipitation of Mn/Co salts in air atmosphere and subsequent crystallization into spinels at mild temperature (180 °C). The approach yields ∼10 nm cobalt–manganese oxide (CoMnO) spinels with independently tunable crystallographic phase (cubic or tetragonal form) and Co/Mn ratio, which is—to our knowledge—the first report of structural control over a wide compositional range of spinel (Co_3−*x*_Mn_*x*_O_4_). In this case, the tetragonal form is extended in a composition range of 1≤*x*≤3, while the cubic symmetry is attainable within 0≤*x*≤2. The selective synthesis also allows the first systematical investigation of structure–performance correlation for CoMnO spinel electrocatalysts. We show that a cubic phase and a high Mn concentration in CoMnO spinels favour intrinsic ORR activity. We demonstrate that the nanocrystalline cubic spinel firmly supported on carbon manifests comparable ORR current and potential, but significantly better stability as compared with the commercial Pt/C catalyst. Furthermore, the spinel-based electrocatalyst is catalytically efficient in air electrodes of rechargeable Zn–air and Li–air batteries.

## Results

### Synthesis and characterization of nanocrystalline spinels

**Figure 1:**
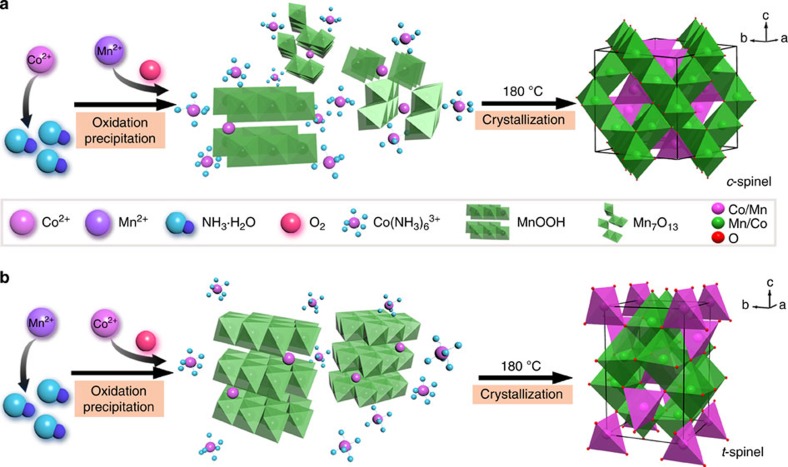
Synthesis of CoMnO spinels. Schematic synthesis of cubic (**a**) and tetragonal (**b**) spinel phases, involving two steps of oxidation precipitation and crystallization.

As schematically shown in Fig. 1a,b, the rational synthesis proceeds through two steps, namely, a room-temperature oxidation precipitation of Mn^2+^/Co^2+^ with NH_3_·H_2_O in air followed by a crystallization of spinel phase with cation redistribution on heating at 180 °C. We achieve phase selection by simply altering the adding order of reactants in the first step. To obtain a cubic spinel, aqueous Mn^2+^ is dripped into a solution containing Co^2+^ and NH_3_·H_2_O; the formation of tetragonal spinel follows reversely dripping Co^2+^ solution in a mixture of Mn^2+^ and NH_3_·H_2_O. We attain compositional control by adjusting the molar ratio of Co/Mn precursors. Three representative compositions (Co:Mn=1:2, 1:1 and 2:1) in both cubic (*c*-) and tetragonal (*t*-) phases afford a batch of six spinel samples, which are designated as *c*-CoMn_2_, *c*-CoMn, *c*-Co_2_Mn, *t*-CoMn_2_, *t*-CoMn and *t*-Co_2_Mn, respectively.

**Figure 2:**
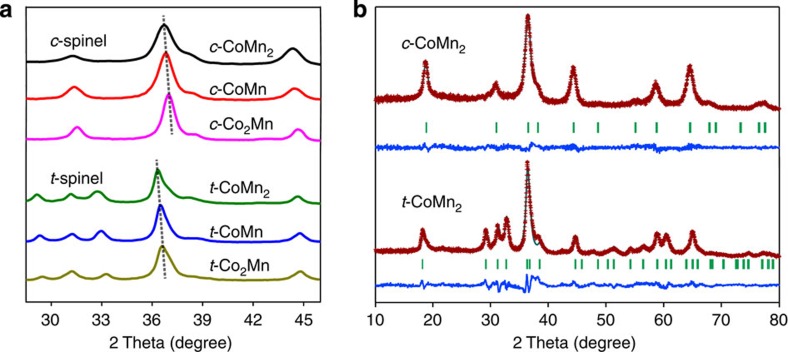
Phases of CoMnO spinels. (**a**) X-ray diffraction patterns of synthesized six spinels and (**b**) Rietveld refined results of *c-, t*-CoMn_2_ spinels. Experimental data, calculated profiles, allowed Bragg diffraction positions and difference curve are marked with red dots, cyan line, vertical bars and blue line, respectively.

Figure 2a,b and Supplementary Fig. 1 display the X-ray diffraction patterns of the as-synthesized samples with the corresponding Rietveld refinement. The peak positions of Co-rich spinels shift to higher angles in both cubic and tetragonal phases (Fig. 2a). Reflection peaks of the cubic series (Fig. 2b; Supplementary Fig. 1a) are indexable to the space group Fd-3m (no.227) and consistent with the standard values of face-centered cubic phase (Joint Committee on Powder Diffraction Standards, JCPDS card no. 23-1237), while the pattern of the tetragonal series (Fig. 2b; Supplementary Fig. 1b) can be readily assigned to body-centered tetragonal spinel (space group I41/amd no. 141, JCPDS card no. 77-0471). The refined profiles match well with the experimental data and suggest the formation of spinel structure with cation or anion deficiency (Supplementary Tables 1–6; Supplementary Note 1). The slightly shifted diffraction peaks and decreased cell parameters of Co-rich oxides are ascribed to substitution of smaller Co^3+^ ions (radius *r*_Co_^3+^=0.545 Å) for Mn^3+^ (*r*_Mn_^3+^=0.645 Å) on the octahedral sites. In comparison with spinels prepared from conventional ceramic routes, the *c*-series have a shrunken lattice, whereas the *t*-series possess larger cell parameters due to the presence of abundant ionic vacancies^13^,^31^.

**Figure 3:**
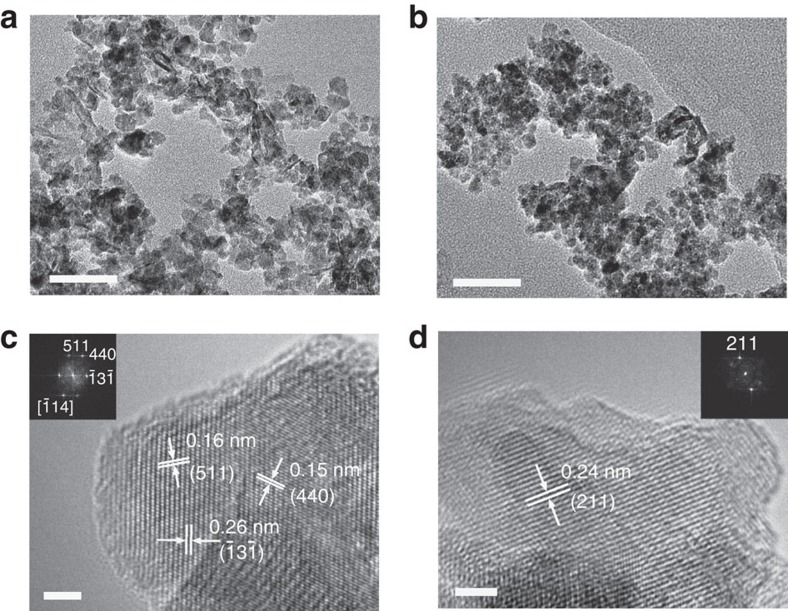
Microstructures of the as-synthesized CoMnO spinels. (**a**,**b**) TEM images showing the particulate shape and small size of *c*-CoMn_2_ (**a**) and *t*-CoMn_2_ (**b**). (**c**,**d)** High-resolution TEM images and FFT patterns (inset) showing the nanocrystalline characteristics of cubic (**c**) and tetragonal (**d**) phases. Scale bars, 50 nm (**a,b**) and 2 nm (**c,d**), respectively.

Typical scanning electron microscopy (SEM) images (Supplementary Fig. 2) show nanoparticulate morphology of the prepared spinel samples. As shown in transmission electron microscopy (TEM) images (Fig. 3a,b; Supplementary Fig. 3), the aggregated particles are composed of small nanocrystals having comparable mass-average particle sizes near 10 nm (Supplementary Fig. 4; Supplementary Note 2). The clear lattice fringes observed in high-resolution TEM and the corresponding fast Fourier transform (FFT) diffraction pattern (Fig. 3c,d) reveal the crystallinity and structural symmetry of the synthesized spinels. The interlayer spacing and well-defined points in FFT patterns coincide with the allowed Bragg diffraction of the corresponding cubic and tetragonal spinels, which agree with the X-ray diffraction analysis. Moreover, energy-dispersive spectra (EDS, Supplementary Fig. 5) and elemental analysis from atomic absorption spectroscopy confirm the compositional ratio of CoMn_2_, CoMn and Co_2_Mn spinel compounds.

**Figure 4:**
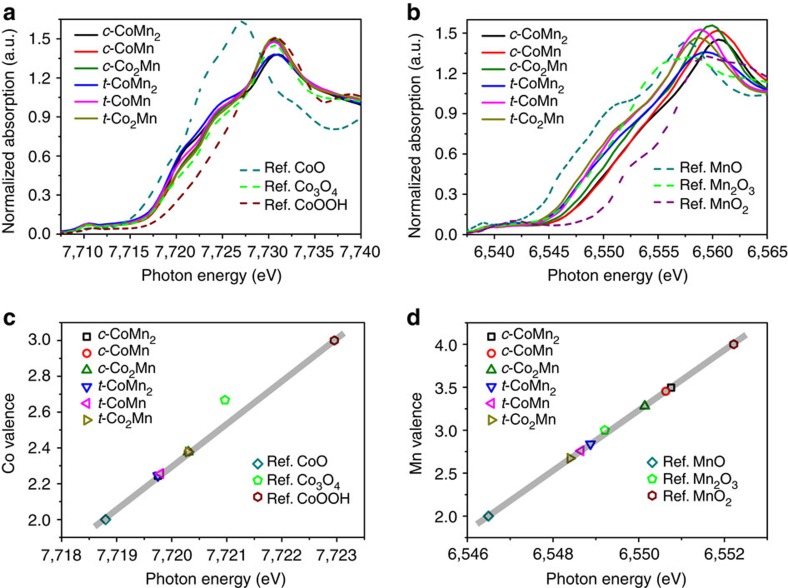
X-ray absorption near-edge structure (XANES) analysis of metal valence. (**a**,**b)** K-edge XANES patterns of Co (**a**) and Mn (**b**) in the synthesized spinels and the reference oxides. (**c**,**d**), Fitted linear relationship between the photon energy and oxidation state of Co (**c**) or Mn (**d**).

Synchrotron X-ray absorption spectroscopy analysis was performed to determine the oxidation states of mixed metals in the synthesized spinels^32^. Figure 4a,b illustrates the Co and Mn K-edge X-ray absorption near-edge structure profiles. The reference cobalt and manganese oxides are also listed and used to establish the linear correlation between the K-edge excitation energy and the metal valence^33^. Accordingly, the mean Co valence was determined to be 2.25 for *c*-CoMn_2_, *t*-CoMn_2_ and *t*-CoMn and 2.38 for *c*-CoMn, *c*-Co_2_Mn and *t*-Co_2_Mn (Fig. 4c). A lower Co valence value tends to appear in oxides with lower Co content, suggesting an increase in the ratio of Co^2+^/Co^3+^ due to substitution of Mn cations^8^. As for Mn (Fig. 4d), the estimated average oxidation states of the cubic group (3.49, 3.45 and 3.28 for *c*-CoMn_2_, CoMn and Co_2_Mn) are obviously higher than those of the tetragonal group (2.84, 2.76 and 2.67 for *t*-CoMn_2_, CoMn and Co_2_Mn). Spectra of Co and Mn L-edge further confirm close Co electronic structure but dissimilar Mn valence (Supplementary Fig. 6). The different Mn oxidation states in the spinels are also verified by their thermal behaviours in air (Supplementary Fig. 7), where the mass loss of cubic phase within a moderate temperature range (250–800 °C) exceeds that of the corresponding tetragonal phase with the same composition. This difference implies more Mn^4+^ reduction to Mn^3+^ and higher Mn valence^34^ in cubic spinels than in tetragonal oxides.

The above results indicate controllable synthesis of cubic/tetragonal spinel phase in an unprecedented wide composition of (Co_3−*x*_Mn_*x*_O_4_, 1≤*x*≤2). Notably, to our knowledge, cubic CoMn_2_O_4_ and tetragonal Co_2_MnO_4_ have never been obtained at mild temperatures. To elucidate the formation mechanism of unusual phase, we have analysed the intermediate species during the synthesis (Supplementary Fig. 8). In preparation of cubic spinels, oxygen from air first oxidizes Co(NH_3_)_6_^2+^ to Co(NH_3_)_6_^3+^ in solution containing Co^2+^ and NH_3_·H_2_O 35. After dripping Mn^2+^, MnOOH forms immediately and is gradually oxidized to cubic Mn_7_O_13_ in Co(NH_3_)_6_^3+^, as evidenced by *ex situ* X-ray diffraction analysis of the precipitate and colour change of the solution (Supplementary Figs 8 and 9). The oxidative precipitation of Mn^2+^ to manganite is well known in alkaline solution exposed to air^36^, while the redox reaction (7MnOOH+5Co(NH_3_)_6_^3+^+5OH^−^→Mn_7_O_13_+5Co(NH_3_)_6_^2+^+6H_2_O, standard Gibbs free energy change -139.53 kJ mol^−1^) is also thermodynamically favourable (Supplementary Note 3). The cubic crystal structure and high Mn valence of intermediate Mn_7_O_13_ (Supplementary Fig. 10) are responsible for the phase speciation of cubic spinel after crystallization on heating. In the synthesis of tetragonal spinels, the main precipitated intermediate is MnOOH, which adopts tetragonal lattice with Mn(III) cations that tend to distort in lower crystallographic symmetry^37^.

Because the present synthesis of CoMnO spinels proceeds at relatively low temperature (180 °C), the product features small particle size and high specific surface area. The obtained *c*-CoMn_2_, *c*-CoMn, *c*-Co_2_Mn, *t*-CoMn_2_, *t*-CoMn and *t*-Co_2_Mn spinels have Brunauer–Emmett–Teller (BET) surface areas of 84, 114, 110, 100, 79 and 73 m^2^ g^−1^ (Supplementary Fig. 11), respectively, which are obviously higher than those of CoMnO spinel powders prepared using the conventional high-temperature ceramic method^29^ (Supplementary Fig. 12). The mild synthesis results in abundant ionic deficiency and low crystallinity, which can be enhanced by annealing (Supplementary Fig. 13). Thermal treatment also induces lower Mn valence as well as larger cell parameter and increased average metal–oxygen bond length in octahedron (Supplementary Table 7). Of particulate note, the unusual *c*-CoMn_2_ and *t*-Co_2_Mn spinels undergo cubic≤tetragonal transition at high temperature (Supplementary Fig. 14), which is consistent with previous report^13^ and suggests that *c*-CoMn_2_ and *t*-Co_2_Mn are metastable. These results demonstrate the successful simultaneous control of crystallographic symmetry and composition of CoMnO spinels.

### Electrocatalytic ORR properties of CoMnO spinels

**Figure 5:**
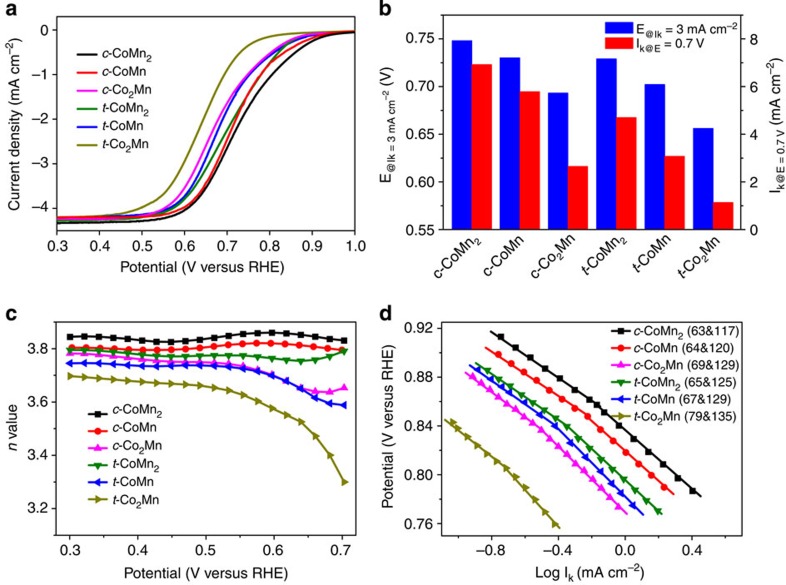
ORR electrocatalytic properties of CoMnO spinels. (**a**), Voltammograms in O_2_-saturated 0.1 M KOH electrolyte by subtracting the voltammograms in Ar. The electrode rotation rate is 900 r.p.m. (**b**), Kinetic current densities at 0.7 V and required potentials for *I*_k_=3 mA cm^−2^. (**c**), Electron transfer number (*n*) at different potentials. (**d**), Tafel plots constructed from the voltammetry data. The slope unit is mV dec^−1^.

The controllable preparation of CoMnO spinels series permits a systematical investigation of their structure–performance relationship towards the oxygen electrocatalysis. Figure 5 shows the ORR catalytic properties of the nanocrystalline spinels supported on rotational glass-carbon electrodes. The polarization profiles (Fig. 5a) display typical ORR current-potential response^21^,^22^,^23^, by subtracting the voltammograms in Ar. There emerges a mixed kinetic diffusion-controlled region at 0.9–0.5 V versus reversible hydrogen electrode and a subsequent oxygen-transport-limited current (*I*_d_), which increases with electrode-rotating rates (Supplementary Fig. 15). The catalytic activities of the spinels can be accessed directly by the onset and half-wave potentials. Evidently, the cubic spinel outperforms the corresponding tetragonal phase having the same composition, while for either cubic or tetragonal structures, the activities decrease with Co:Mn ratio, following an order of CoMn_2_>CoMn>Co_2_Mn. The different performance can be also viewed from the required potentials to reach a given kinetic current (*I*_k_) or the *I*_k_ values at a given potential (Fig. 5b).

Further evaluation of the ORR catalytic behaviours is performed by determining the transferred electron number (*n*), and yield of peroxide species from the disk and ring currents recorded on rotational disk–ring electrodes (Fig. 5c; Supplementary Fig. 16; Supplementary Note 4). Below 0.55 V, *n* is above 3.60, which coincides with the results based on Koutecky–Levich^8^,^28^ plots (Supplementary Fig. 15; Supplementary Note 5) and suggests an apparent quasi-4e^−^ ORR pathway. A comparison of determined *n* values for six nanocrystalline spinels demonstreates the superiority of cubic phase and high Mn content. In addition, two Tafel slopes observed at high and low overpotentials imply similar reaction mechanism and rate-determining step for all spinels (Fig. 5d), in accordance with previously reported manganese oxide electrocatalysts^38^. Again, the activities of different spinels are distinguishable by the overpotential and kinetic current density. It is deserved to note that the ORR activities of synthesized nanocrystalline CoMnO spinel surpass that of the counterparts prepared using high-temperature ceramic method (Supplementary Fig. 17); this superiority could be mainly ascribed to the smaller crystalline size and higher specific surface areas. Besides, the obtained spinel renders respectable catalytic capability in neutral (KCl solution) or nonaqueous (ether-based electrolyte) media (Supplementary Fig. 18).

Supplementary Table 8 summarizes the ORR electrocatalytic characteristics (for example, onset and half-wave potentials, transferred electron number and kinetic currents normalized by mass or specific surface areas) of the spinel series, indicating structure and composition co-dependent behaviour. Specifically, cubic phase and higher Mn/Co ratio are more favourable in intrinsic activity. In alkaline media, the ORR electrocatalysis on transition metal oxides is proposed to involve multistep steps of O_2_ adsorption, formation of an HO_2_^−^ intermediate and further reduction or decomposition of peroxide to OH^−^ ions^28^. These processes are mediated by electron transfer and redox reactions of metal-containing species, and strongly related to the number of available active sites and the surface oxygen adsorption ability that can be tuned by electronic structure^38^,^39^. Following this mechanistic consideration, the structure–property correlation of CoMnO spinels can be understood as follows. First, cubic spinel surfaces provide more catalytic sites number and bind O_2_ more strongly than the tetragonal phase, as previously demonstrated^29^. The slightly stronger interaction between cubic spinel and adsorbed oxygen is verified here by X-ray photoelectron spectroscopy (XPS, Supplementary Fig. 19). Second, the cubic phases feature a higher average oxidation state of Mn (>3), which exists in a mixed form of Mn^3+^ and Mn^4+^ cations. The presence of multivalent 3*d* metals would benefit electron conduction (by hopping) and charge transfer (through redox reactions), thus favouring the electrocatalysis^40^. The electrical conductivity of neat *c*-CoMn_2_ is 1.01 S m^−1^, a value at least three orders of magnitude higher than that of the binary Co_3_O_4_ and Mn_3_O_4_ spinels (Supplementary Fig. 20). Third, the composition-dependent kinetics is interpretable by the factor that Co(II) is less active than Mn species^8^. Furthermore, based on O K-edge X-ray absorption spectroscopy analysis (Supplementary Fig. 6), we find a moderate affinity between surface metal cation and absorbed oxygen-containing species in both *c*-CoMn_2_ and *t*-CoMn_2_ spinels, which would facilitate the rate-limiting steps of hydroxide/peroxide displacement and regeneration during the ORR^28^.

### Application of carbon-supported CoMnO spinels

**Figure 6:**
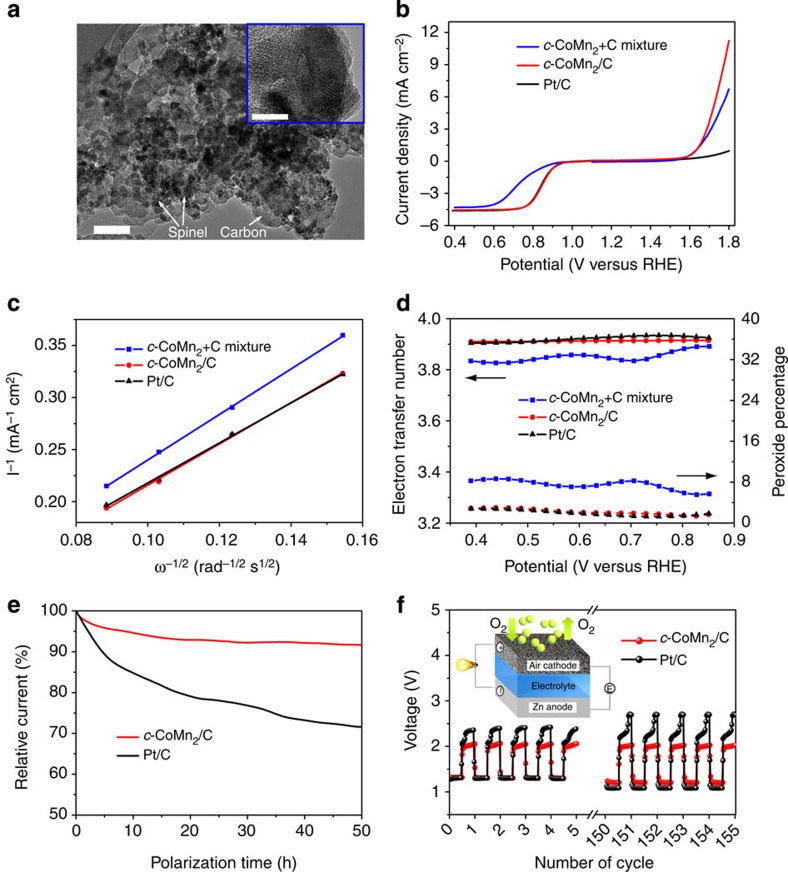
Carbon-supported CoMnO spinel for ORR/OER and metal–air batteries. (**a**), TEM image of prepared *c*-CoMn_2_/C hybrid (scale bar, 50 nm). Inset shows firm anchoring of a single spinel nanoparticle on carbon (scale bar, 10 nm). (**b**), Comparison of ORR and OER activities of *c*-CoMn_2_+C mixture, *c*-CoMn_2_/C hybrid and Pt/C catalyst in 0.1 M KOH solution. The rotation rate is 900 r.p.m. during the ORR. (**c**), K–L plots at 0.65 V. (**d**), Electron transfer number and peroxide percentage generated at various potentials. (**e**), Chronoamperometric responses of *c*-CoMn_2_/C and Pt/C at 0.8 V in O_2_-saturated 0.1 M KOH. (**f**), Performance of rechargeable Zn–air cells based on *c*-CoMn_2_/C and Pt/C catalysts at a cycling rate of 10 mA cm^−2^ and a duration of 400 s per cycle. Inset schematically depicts the structure of assembled rechargeable Zn–air cells.

Our one-pot synthesis offers a simple, general and *in situ* approach to fabricate mixed-metal oxide supported on conducting substrate composite materials with high electronic conductivity and uniformly distributed active sites. Here we show an example of the hybrid (Supplementary Fig. 21) comprising the most active *c*-CoMn_2_ spinel and Carbot Vulcan XC-72 carbon (a widely used electrocatalyst support^29^), which is abbreviated as *c*-CoMn_2_/C. TEM image (Fig. 6a) of the composite reveals that *c*-CoMn_2_ nanoparticles are uniformly dispersed on the carbon support with firm attachment. The composition of the hybrid can be easily adjusted and a ∼30 wt% spinel is found to attain the best ORR activity (Supplementary Fig. 22).

The as-synthesized *c*-CoMn_2_/C composite delivers significantly enhanced ORR and OER performance as compared with the physical mixture of two components (Fig. 6b). More surprisingly, the ORR activity of the hybrid rivals that of the benchmark Pt/C catalyst (30 wt% Pt nanoparticles supported on Vulcan XC-72 carbon, Supplementary Fig. 23); for the OER the former far exceeds the latter. The similar fitted K–L plots (Fig. 6c) and determined electron transfer number and percentage of peroxide (Fig. 6d) also demonstrate the comparable catalytic performance between the hybrid and Pt/C. In addition, the calculated ORR turnover frequency (Supplementary Note 6) at 0.80 V is 0.69, 1.07 and 0.07 s^−1^ for Pt/C, *c*-CoMn_2_/C, and the physical mixture, respectively. The key performance parameters such as onset potential, half-wave potential, kinetic current density and electron transfer number of *c*-CoMn_2_/C are among the most active non-noble metal electrocatalysts reported so far^8^,^27^,^28^,^29^,^41^ (Supplementary Table 9). Electrochemical impedance spectroscopy (Supplementary Fig. 24) shows much lower charge transfer resistance on *c*-CoMn_2_/C than on *c*-CoMn_2_+C mixture, which is likely an attribution to the faster ORR kinetics of the *in situ* generated hybrid. Apart from high activity, *c*-CoMn_2_/C exhibits remarkable catalytic stability. In a continuous chronoamperometric period of 50 h, the retention of ORR current on *c*-CoMn_2_/C sustains >91.5%, significantly higher than that of Pt/C (71.6%) (Fig. 6e). The morphology of the hybrid remains almost unchanged even after repeated 12,000 cyclic voltammograms, whereas Pt nanoparticles agglomerate severely in Pt/C (Supplementary Fig. 25), indicating considerable structural robustness of *c*-CoMn_2_/C. The superior performance of the hybrid possibly arises from several factors: (1) the intrinsic high activity of *c*-CoMn_2_ spinel (Supplementary Table 8); (2) the high dispersion, small size of nanocrystalline spinel; and (3) the strong coupling effect between the spinel oxide and the carbon substrate functionalized with nitrogen groups, as evidenced by XPS results (Supplementary Fig. 26). The firm contact of spinel and carbon in the *in situ* fabricated hybrid also favours enhanced electrical conductivity in comparison with the physical mixture (Supplementary Fig. 20).

The superb electrocatalytic performance of synthesized *c*-CoMn_2_/C has motivated us to investigate its applicability in metal–air battery devices. The assembled primary Zn–air cells exhibit stable galvanostatic discharge curves, giving a high energy density of ∼650 Wh kg^−1^ at 10 mA cm^−2^ normalized to consumed Zn anode (Supplementary Fig. 27). More interestingly, the constructed cells show exceptional rechargeability at a shallow discharge/charge state (Fig. 6f). Due to the superior bifunctinal ORR/OER capability and durability, the *c*-CoMn_2_/C composite electrocatalyst enables lower discharge/charge overpotential and more stable voltage plateau on cycling, as compared to that of Pt/C. For *c*-CoMn_2_/C-based cell, the average voltage of discharge platform decreases by 8.5% after 155 cycles, half that of Pt/C (17.6%). The variation of charging potential for *c*-CoMn_2_/C electrode is negligible, whereas the Pt/C-based cell suffers a increase of 14.4%. Furthermore, the spinel-based cathode applied in lithium–air cells delivers high capacity and encouraging cyclability up to dozens of cycles (Supplementary Fig. 28). These results suggest that the *c*-CoMn_2_/C nanocomposite is an effective, yet durable catalyst for catalysing dual ORR and OER in rechargeable metal–air batteries.

## Discussion

We have synthesized nanocrystalline cobalt–manganese spinel oxides with simultaneously controllable phase and composition using a facile oxidation–precipitation and crystallization route. The synthesis allows tuning the cubic and tetragonal crystallographic symmetry of spinel over an unprecedented wide compositional range (Co_3−*x*_Mn_*x*_O_4_, 1≤*x*≤2) at mild temperature and room atmosphere. The obtained spinel family is featured by variable metal valence, small particle size, high specific surface areas and abundant ionic defects. It deserves to note that this solution-based synthetic strategy can be extended to the preparation of other technologically important nanocrystalline spinel oxides such as NiCo_2_O_4_, FeCo_2_O_4_, ZnCo_2_O_4_ and ZnMn_2_O_4_ at temperatures below 200 °C (Supplementary Fig. 29), although the structural symmetry control of these spinels has not been attained possibly owing to the limit of valence tunability and the strong lattice-site preference of Ni, Fe and Zn relative to Co and Mn.

When employed as electrocatalysts for the ORR in alkaline media, the as-synthesized spinel nanocrystallines outperform the high-temperature powders. The intrinsic electrocatalytic activity of CoMnO family correlates with the crystal phase, Co/Mn composition and surface valence value that affect the extent of O_2_ activation and number of available active sites. Furthermore, the solution-based synthesis at mild temperatures enables *in situ*, firmly dispersing ultrafine spinel nanoparticles on conducting substrates such as carbon. The optimized *c*-CoMn_2_/C hybrid manifests similar ORR performance and superior catalytic durability to that of the counterpart Pt/C catalyst. The *c*-CoMn_2_/C nanocomposite is also active for the OER, rendering bifunctional electrocatalytic capability. These results would shed light on rational design and preparation of spinel oxides and broaden their applications, for example, as efficient catalysts for oxygen-relevant energy storage technologies including rechargeable metal–air batteries and regenerative fuel cells.

## Methods

### Synthesis

In a typical synthesis of cubic nanocrystalline CoMn_2_ spinel, 4 ml aqueous ammonia (25 wt%) was first dropped into 5 ml 0.2 mol l^−1^ Co(NO_3_)_2_ solution under constant stirring at room temperature. Then, 10 ml of 0.2 mol l^−1^ Mn(NO_3_)_2_ solution was added to the mixture, which was stirred for 120 min. Afterwards, the mixture was evaporated and nitrates were fully decomposed by heating at 180 °C for 40 min, yielding cubic CoMn_2_. Similarly, cubic CoMn and Co_2_Mn samples were obtained by adopting the corresponding stoichiometric amount of cobalt and manganese sources, respectively. In preparation of tetragonal CoMn_2_, 4 ml aqueous ammonia (25 wt%) was dropped into 10 ml 0.2 mol l^−1^ Mn(NO_3_)_2_ solution, subsequently followed by addition of 5 ml 0.2 mol l^−1^ Co(NO_3_)_2_ aqueous solution, water evaporation and heating. Likewise, tetragonal CoMn and Co_2_Mn spinels were synthesized by adjusting the Co:Mn molar ratio. The spinel/carbon hybrid was obtained by simply dispersing desired amount of carbon powders in the aqueous Co/Mn salt solution.

### Characterization

Powder X-ray diffraction patterns were collected on a Rigaku MiniFlex600 X-ray diffractometer with Cu *Kα* radiation. The X-ray diffraction data was refined by the RIETAN-2,000 Rietveld refinement program^42^. SEM images were taken with a JEOL JSM-7500F microscope (operating voltage, 5 kV) equipped with an EDS analyzer. TEM and high-resolution TEM imaging was obtained on Philips Tecnai G2 F20 (acceleration voltage, 200 kV). Chemical composition was determined by EDS and atomic absorption spectrometry (AAS, Hitachi 180-90 spectrophotometer). Surface areas were calculated from the N_2_ adsorption/desorption isotherms at 77 K on BELsorp-Mini. Thermogravimetric analysis was carried out on a Netzsch STA 449 F3 Jupiter analyzer. XPS was performed by a Perkin Elmer PHI 1,600 ESCA system. The X-ray absorption near-edge structure spectra were collected on BL14W1 beamline of Shanghai Synchrotron Radiation Facility (SSRF) and analysed with software of Ifeffit Athena.

### Electrochemical test

Electrochemical tests were performed using a standard three-electrode electrochemical cell comprising a saturated calomel reference electrode, a platinum foil counter electrode and a glass-carbon working electrode coated with catalyst. The fabrication of working electrode followed similar procedures described previously^40^. Typically, a mixture of 1.5 mg spinels and 3.5 mg carbon powder (Vulcan XC-72) or 5 mg *c*-CoMn_2_/C hybrid was dispersed in solvent containing 0.5 ml water, 0.5 ml isopropyl alcohol and 17.5 μl neutralized Nafion solution (5 wt%, Sigma-Aldrich). After thorough sonication, 4.6 μl of the formed catalyst ink was pipetted on the glassy carbon electrode, which was air-dried to afford a mass loading of ∼0.054 mg_oxide_ cm^−2^. The electrolyte was 0.1 M aqueous KOH, 0.1 M KCl and 1.0 M lithium bis(trifluoromethane) sulfonamide (LiTFSI) in tetraethylene glycol dimethyl ether (TEGDME) in the case of alkaline, neutral and aprotic media, respectively. The electrolyte was purged with O_2_ for 30 min before each measurement and subjected to O_2_ flow during the tests. Electrochemical data were recorded at room temperature on a PARSTAT 263 A workstation accompanied with a model 636 system (AMETEK). The voltammetry was collected at a potential scan rate of 5 mV s^−1^. The polarization voltammograms in O_2_-saturated electrolyte have been corrected by subtracting the background current recorded in Ar-saturated electrolyte. All potentials were calibrated with reference to standard reversible hydrogen electrode. Electrochemical impedance spectra were conducted on PARSTAT 4,000 (AMETEK) with a frequency range of 100 kHz–100 mHz. The Li–air and Zn–air cells were assembled with a Li or Zn anode, a spinel catalyst-based cathode and a LiTFSI/TEGDME or KOH electrolyte. Battery performance testing was conducted on a LAND-CT2001A system at room temperature.

## Additional information

**How to cite this article**: Li, C. *et al*. Phase and composition controllable synthesis of cobalt manganese spinel nanoparticles towards efficient oxygen electrocatalysis. *Nat. Commun.* 6:7345 doi: 10.1038/ncomms8345 (2015).

## Supplementary Material

Supplementary Information Supplementary Figures 1-29, Supplementary Tables 1-9, Supplementary Notes 1-6, Supplementary Methods and Supplementary References.
