# Supplementary material for: Phase and composition controllable synthesis of cobalt manganese spinel nanoparticles towards efficient oxygen electrocatalysis
Source: Nat Commun. 2015 Jun 4;6:7345. doi: 10.1038/ncomms8345 (PMC4468846; doi:10.1038/ncomms8345)
Supplement: Supplementary Information — Supplementary Figures 1-29, Supplementary Tables 1-9, Supplementary Notes 1-6, Supplementary Methods and Supplementary References. [file ncomms8345-s1.pdf]

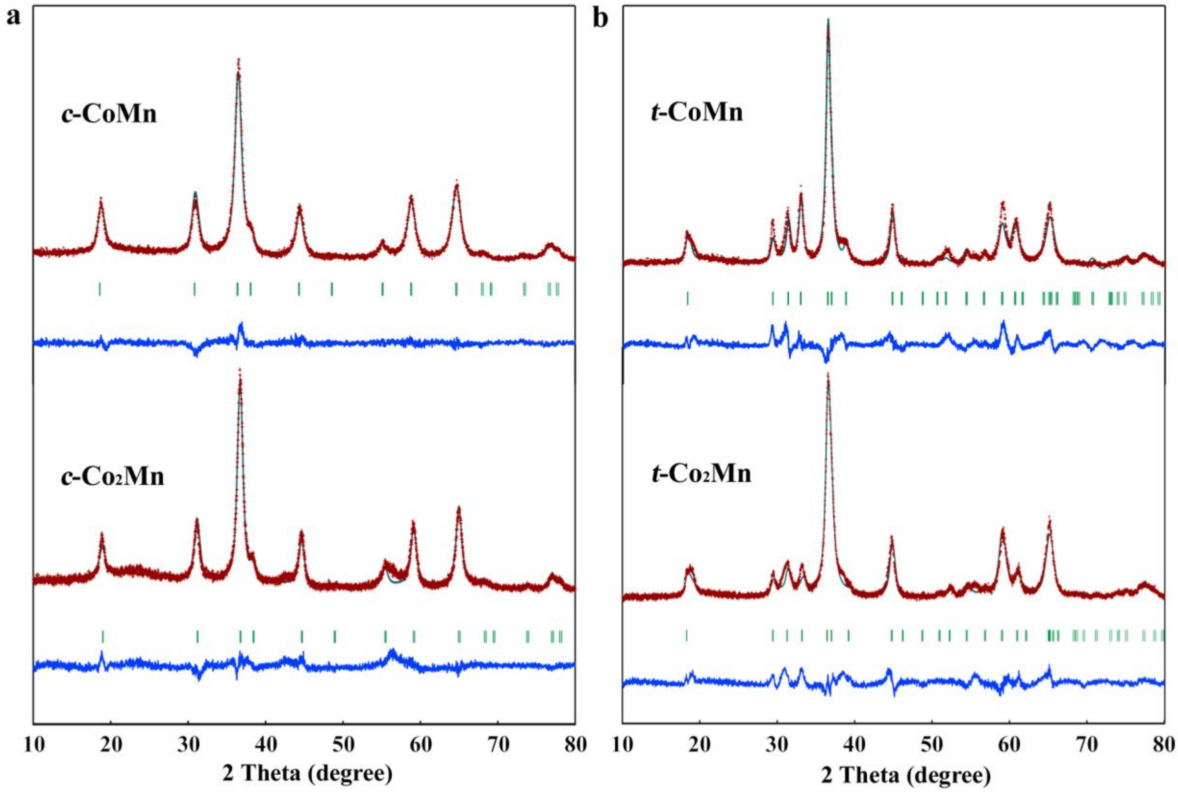

Supplementary Figure 1 | Rietveld refined XRD patterns. Experimental data, calculated profiles, allowed Bragg diffraction positions and difference curve are marked with red dots, cyan line, vertical bars and blue line, respectively. Results are summarized in Supplementary Table 2,3,6,7. Additional discussion is given in Supplementary Note 1.

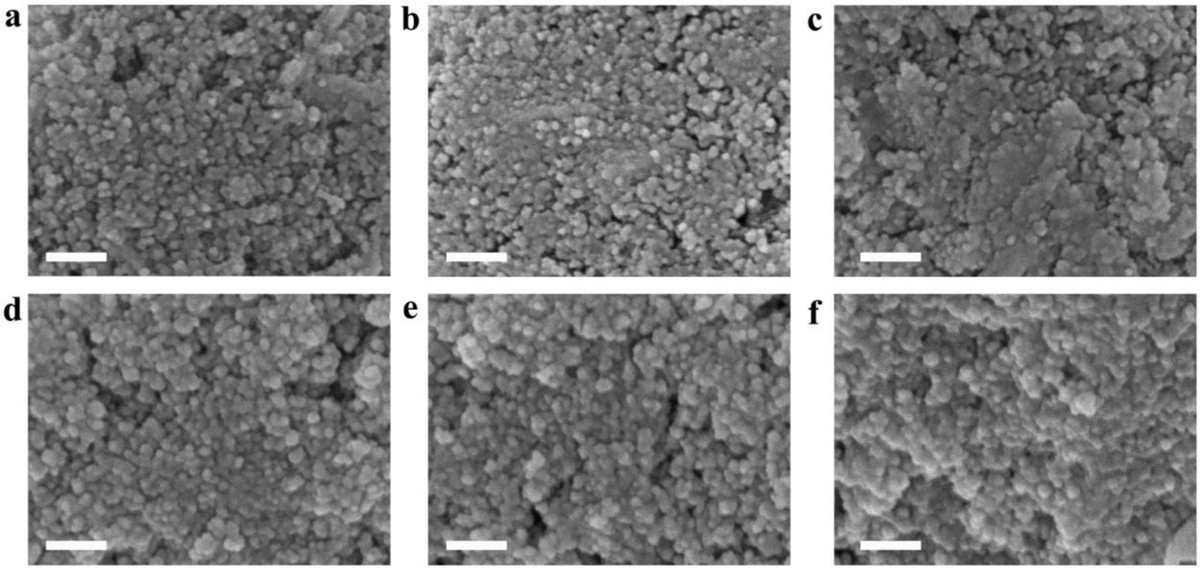

Supplementary Figure 2 | SEM characterization. SEM images of synthesized spinels: (**a**) *c*-$CoMn_2$, (**b**) *c*-CoMn, (**c**) *c*-Co_2Mn, (**d**) *t*-$CoMn_2$, (**e**) *t*-CoMn, and (**f**) *t*-Co_2Mn. All scale bars are 100 nm.

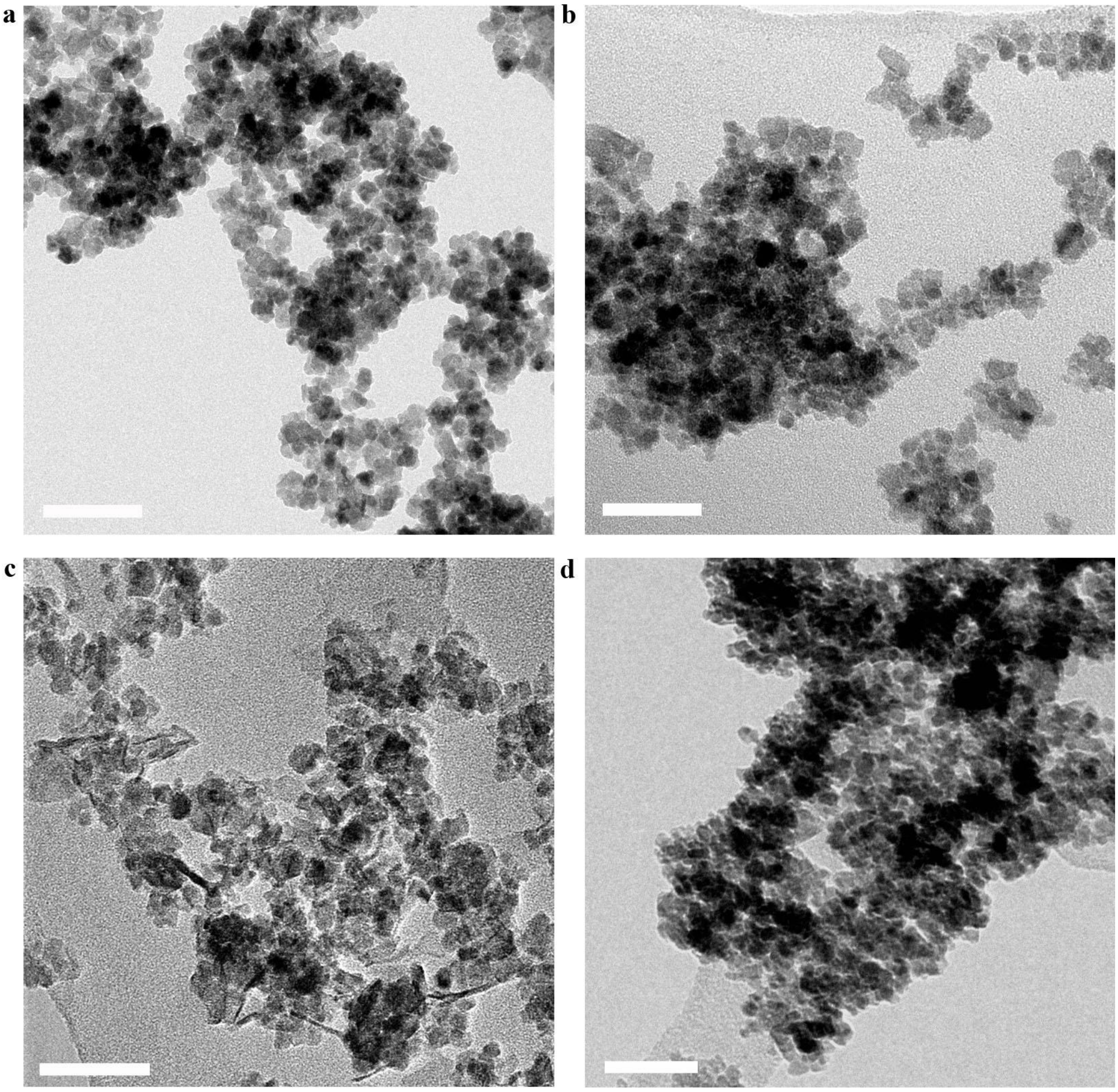

Supplementary Figure 3 | TEM imaging. TEM images of synthesized spinels: (**a**) *c*-CoMn, (**b**) *c*-Co_2Mn, (**c**) *t*-CoMn and (**d**) *t*-Co_2Mn. All scale bars are 50 nm.

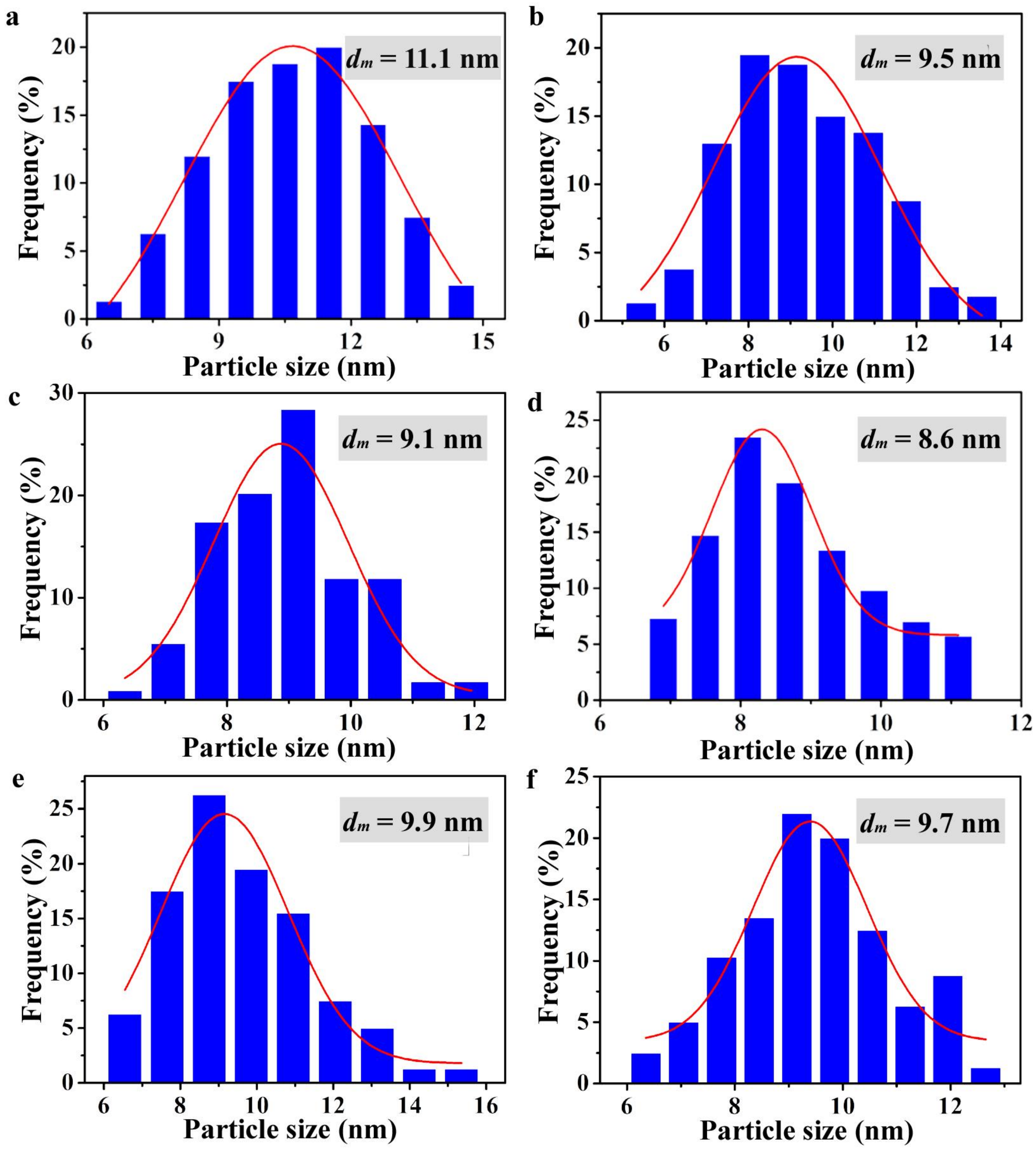

Supplementary Figure 4 | Particle size analysis. Size distribution of (**a**) *c*-$CoMn_2$, (**b**) *c*-CoMn, (**c**) *c*-Co_2Mn, (**d**) *t*-$CoMn_2$, (**e**) *t*-CoMn and (**f**) *t*-Co_2Mn. The statistical data are based on the TEM imaging. Calculation of mass-average particle size (d_m) is described in Supplementary Note 2.

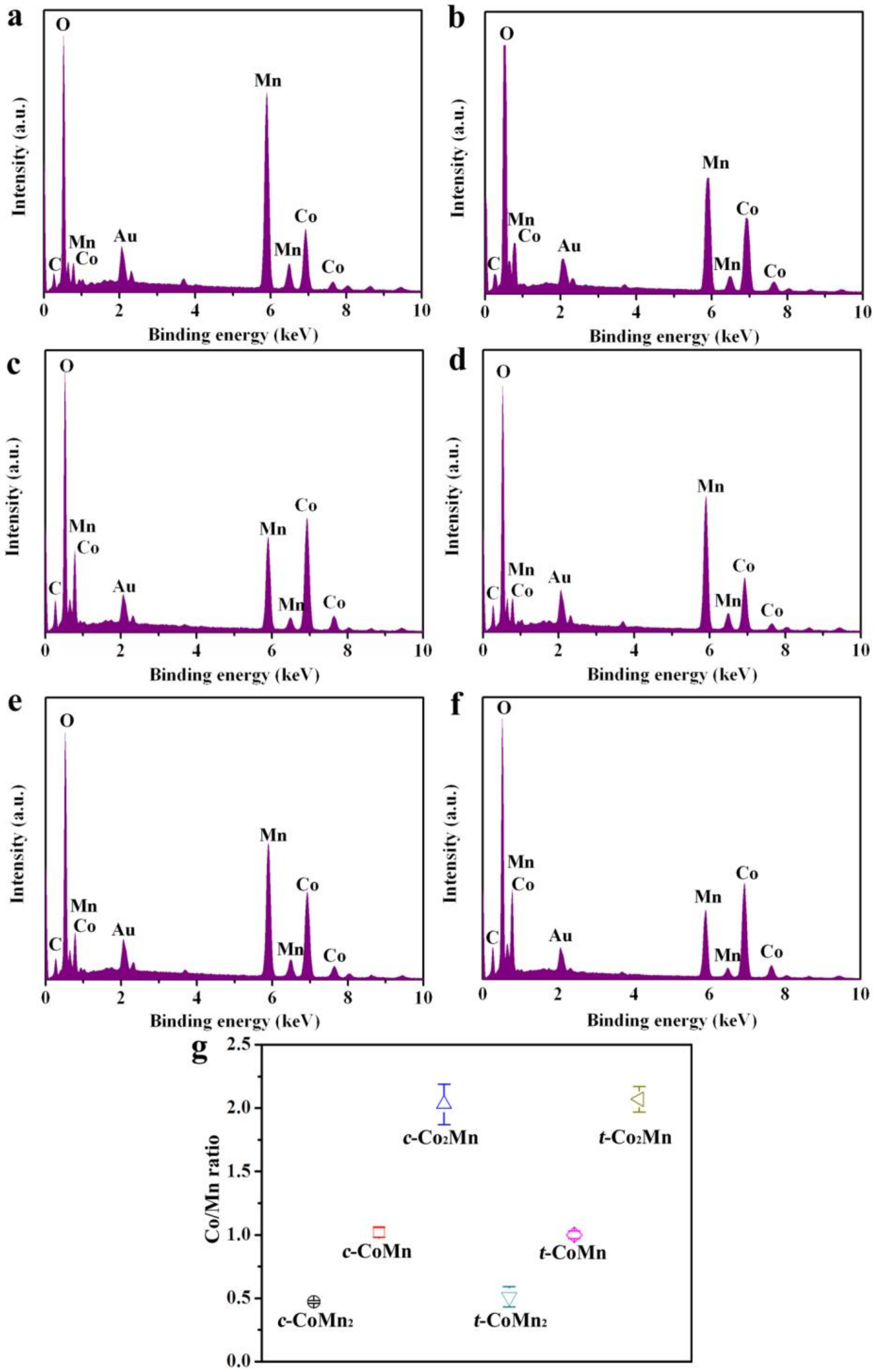

Supplementary Figure 5 | EDS data of CoMnO spinels. EDS analysis of synthesized six samples: (**a**) *c*-$CoMn_2$, (**b**) *c*-CoMn, (**c**) *c*-Co_2Mn, (**d**) *t*-$CoMn_2$, (**e**) *t*-CoMn and (**f**) *t*-Co_2Mn. (**g**) Co/Mn ratio in prepared CoMnO spinels determined by EDS. Error bars represent standard deviation of at least three measurements. The EDS elemental results confirm the corresponding Co/Mn ratio of each synthesized oxide and are well consistent with the nominal values and the results determined from AAS.

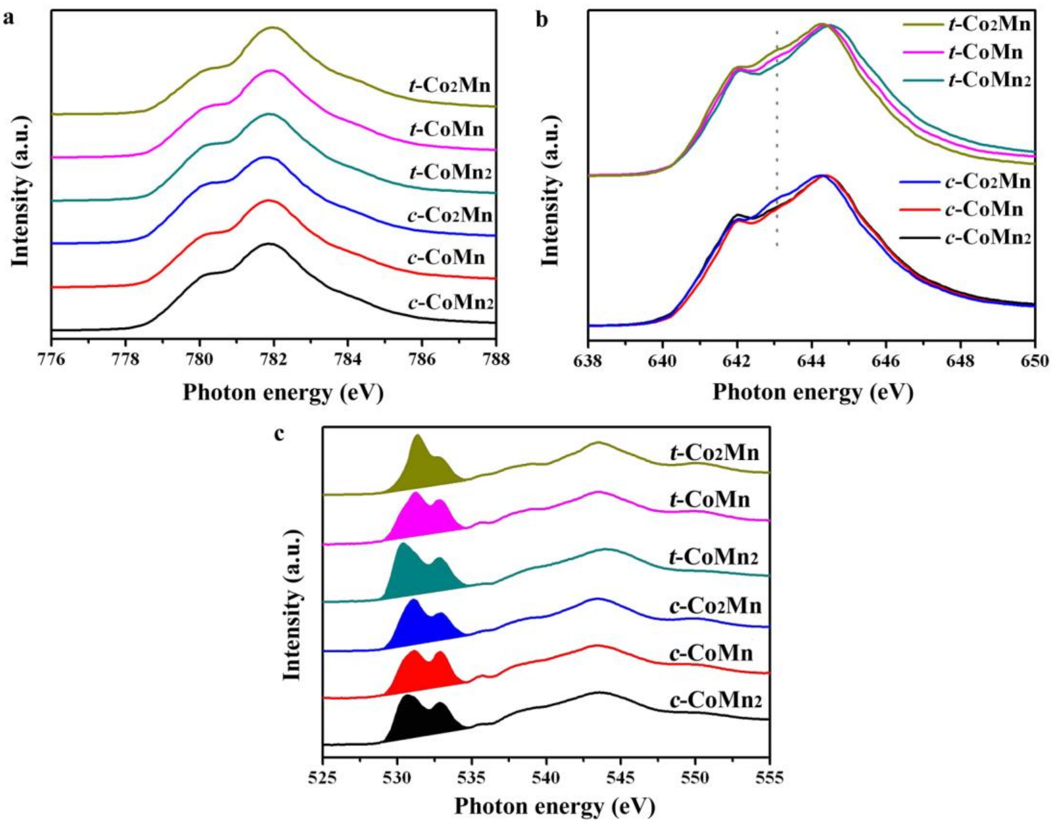

Supplementary Figure 6 | Soft X-ray Absorption characterization. L_{III} peaks of Co (**a**), Mn (**b**) L-edge and O K-edge (**c**) spectra. The shoulders on the lower-energy side of Co L-edge L_{III} peaks spectra (**a**) are due to Co 2p-3d electrostatic interaction and the crystal field of tetrahedral symmetry[1,2], indicating that the cobalt ions preferably occupy the tetrahedral sites. The increasing shoulders pointed by dot line in Mn L-edge L_{III} peaks (**b**) suggest decreased Mn average valence[1], in agreement with Mn K-edge spectra. The O K-edge peaks (**c**) at 531 eV of *c*-CoMn, *c*-Co_2Mn, *t*-CoMn and *t*-Co_2Mn shift towards higher energy region as compared with *c*-$CoMn_2$ and *t*-$CoMn_2$. This demonstrates that, in the crystal lattice of *c*-$CoMn_2$ and *t*-$CoMn_2$, fewer cobalt ions are present at octahedral sites[3], in agreement with XRD refined results. Moreover, peak at 533 eV in O K-edge (**c**) reveals the hybridization state of metal $3dz^2$ and external O species π* orbits[3,4]. Particularly, *c*-$CoMn_2$ and *t*-$CoMn_2$ with moderate peak intensity among the cubic and tetragonal series balance the rate-limiting steps of O_2^{2-}/OH^- displacement and OH^- regeneration on the surface of transition-metal ions during ORR, thus displaying the highest ORR catalytic capability (see electrochemical results), in accordance with previous report[5].

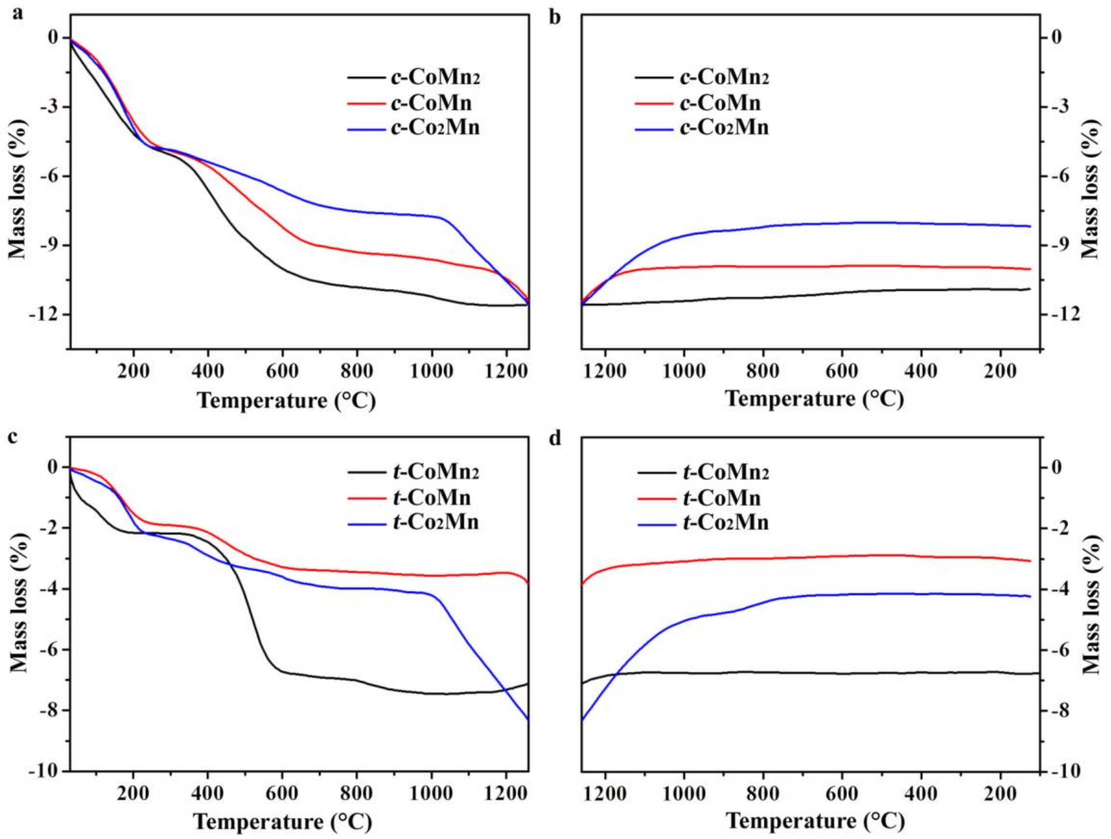

Supplementary Figure 7 | Thermal analysis. Mass loss versus temperature recorded on cubic (**a**, **b**) and tetragonal (**c**, **d**) spinels with three compositions upon heating from 25 °C to 1260 °C and cooling from 1260 °C to 100 °C in air flow. Heating/cooling rate: 10 °C min^{-1}. The slow mass loss before 250 °C is caused by the removal of water and hydroxy groups probably adsorbed on the surface of the oxide particles. The continuous mass loss in TG curves from 250 °C to 800 °C is ascribed to the gradual removal of oxygen, while the progressive reduction from Mn^{4+} and Co^{3+} to Mn^{3+} and Co^{2+} occurs[6,7]. Of particulate note, the mass loss of cubic series oxides within this temperature range is larger than that of the corresponding tetragonal phase with same composition, suggesting more Mn^{4+} contained in cubic spinels and thus confirming their higher Mn valence values than those of tetragonal oxides. From 800 °C up to the limit temperature (1260 °C), the rapid mass loss around 1000 °C was assigned to the formation of rock-salt-type manganese cobalt oxides along with O_2 evolution[7]. Upon cooling, a partly reversibly mass gain takes place, which is considerably smaller than the mass loss due to the formation of CoO.

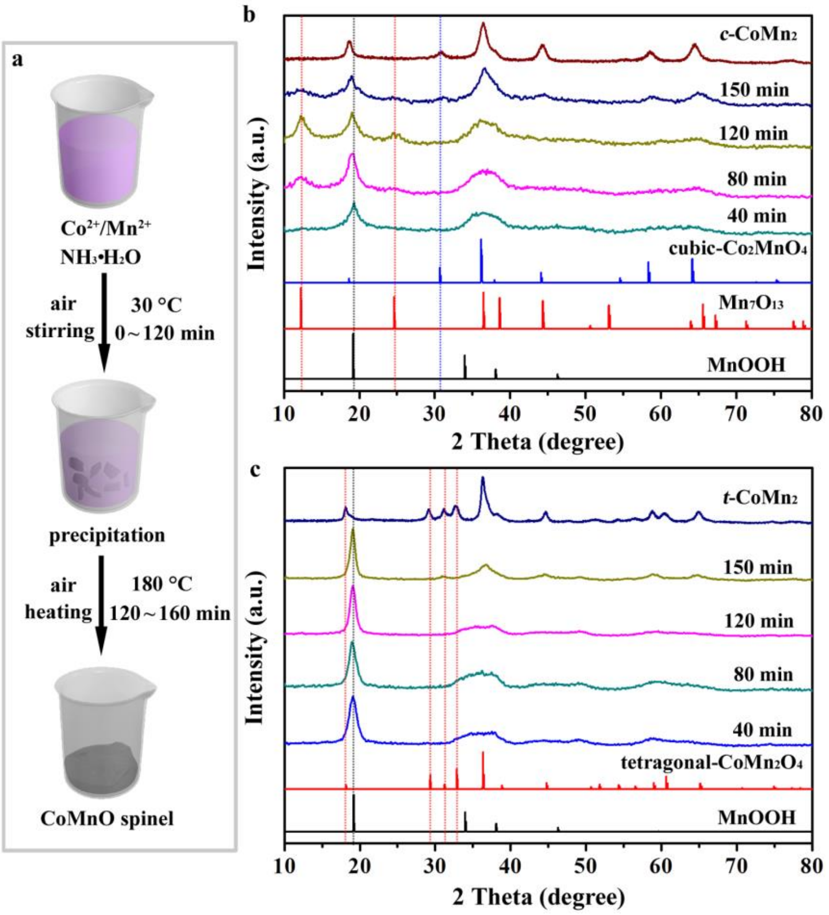

Supplementary Figure 8 | Characterizations of intermediates. (**a**) Scheme for experimental operation and (**b**,**c**) ex-situ XRD patterns of intermediates during the synthesis process for (**b**) cubic and (**c**) tetragonal spinels. Standard oxides of MnOOH (JCPDS no. 18-0804), Mn_7O_{13} (JCPDS no. 23-1239), cubic Co_2MnO_4 (JCPDS no. 23-1237), tetragonal $CoMn_2O_4$ (JCPDS no. 77-0471) and final products (*c*, *t*-$CoMn_2$) are also listed for comparison. As shown in (a), 0 min refers to the time when Co^{2+} or Mn^{2+} was initially mixed with $NH_3{\cdot}H_2O$ solution. After stirring for 120 min at 30 °C (0–120 min), the temperature of the system was elevated to 180 °C. The CoMnO spinels were obtained after continued crystallization process (120–160 min) at 180 °C. Intermediates at different reaction time (40, 80, 120, 150 min) were collected for XRD analysis. Representative peaks of standard samples are marked with dotted lines. Apart from MnOOH, Mn_7O_{13} appears during the preparation process of cubic spinel whereas only manganite forms in the case of tetragonal phase. The cubic and tetragonal phases of Mn_7O_{13} and MnOOH and the different Mn valence are responsible for the formation of final spinel phases.

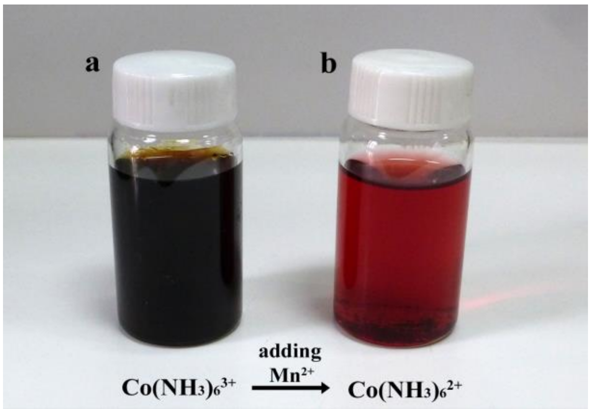

Supplementary Figure 9 | Photos of cobalt-ammonium solution collected during the preparation of cubic spinels. (**a**) aqueous ammonia solution after adding Co^{2+} and stirring for 2 h in air; (**b**) the filtered aqueous solution of cobalt-ammonium after adding Mn^{2+} under Ar. In air atmosphere, O_2 could oxidizes $Co(NH_3)_6^{2+}$ to $Co(NH_3)_6^{3+}$ solution with almost black color, agreeing with previous report[8]. After adding Mn^{2+}, MnOOH was rapidly formed at alkaline condition. Owing to the high redox potential, $Co(NH_3)_6^{3+}$ can oxidize MnOOH to Mn_7O_{13}, with the solution color changing from black to wine red. The transformation of MnOOH to Mn_7O_{13} is thermodynamically favorable (Supplementary Note 3).

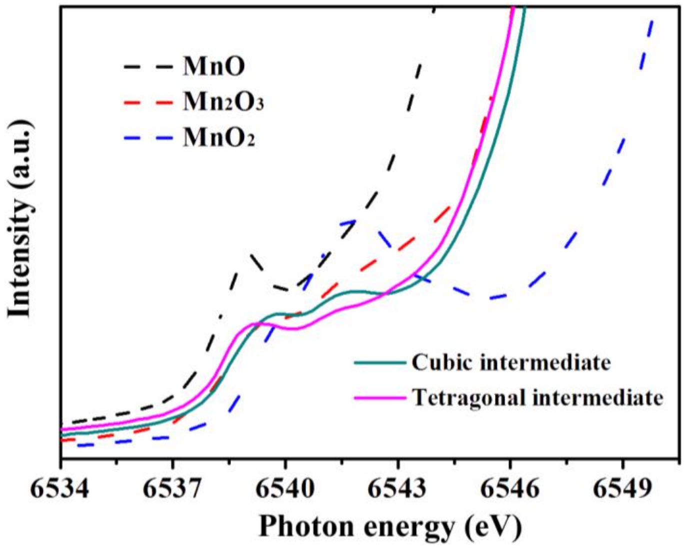

Supplementary Figure 10 | XAS characterization of intermediates. Pre-edge peaks in K-edge XANES spectra of the immediate solids for preparing cubic and tetragonal spinels at 120 min. Profiles of MnO, Mn_2O_3 and MnO_2 standard oxides are listed for comparison. Spectral details in the pre-edge curves provide valuable information about the oxidation state of Mn absorbers. A small hump observed in the pre-edge region is assigned to Mn 1s $\rightarrow$ 3d transition. Noticing pre-edge peak energies of our samples are similar to standard sample of Mn_2O_3. Another positive peak appears for cubic intermediate and a slightly peak negatively shifts for tetragonal intermediate, indicating that the average Mn valence of intermediate solid for cubic spinel locates between Mn(III) and Mn(IV) whereas that of tetragonal intermediate is between Mn(III) and Mn(II).

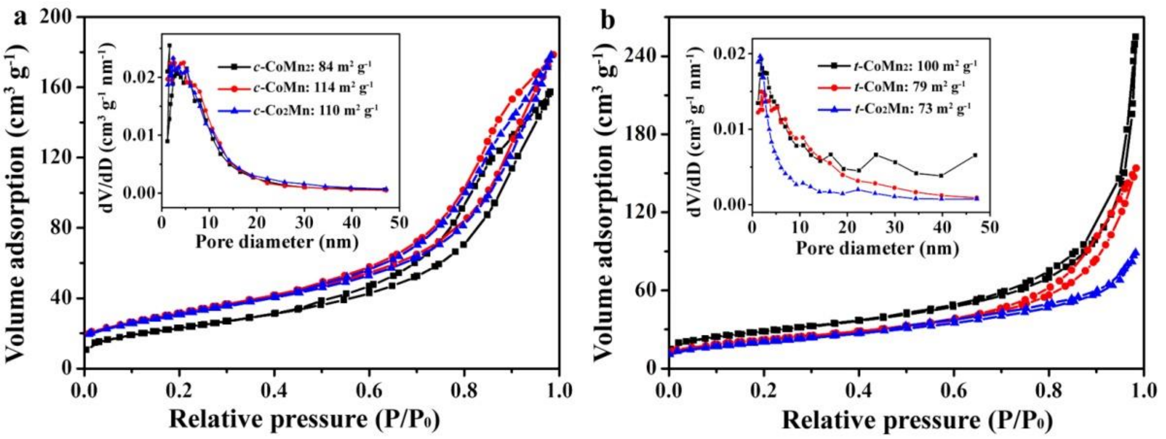

Supplementary Figure 11 | Nitrogen adsorption and desorption isotherms measured at 77 K. The Brunauer-Emmett-Teller (BET) surface areas of current spinel samples are much larger than those of cubic and tetragonal spinel particles prepared through conventional high-temperature method[9]. In addition, the corresponding pore size distribution (inset) determined by the Barrett-Joyner-Halenda (BJH) shows the pore diameter centered below 20 nm, verifying the porous structure.

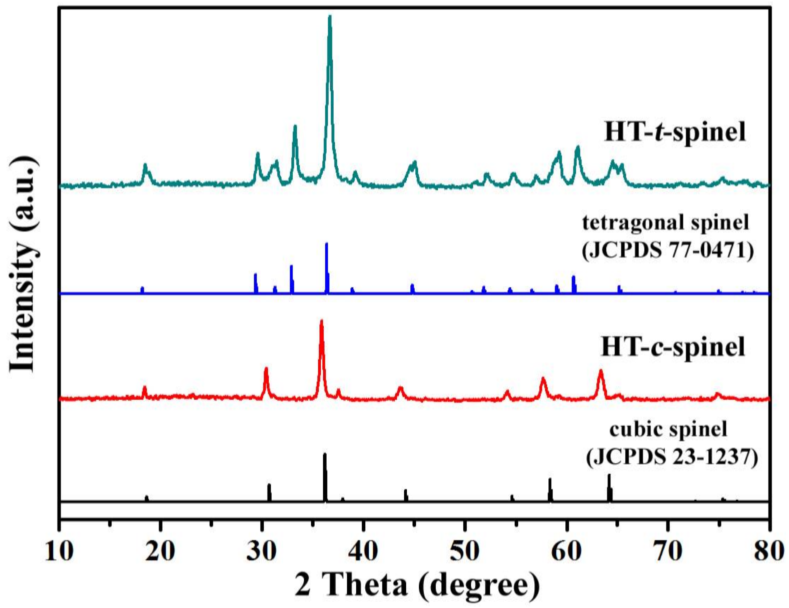

Supplementary Figure 12 | XRD profiles of the synthesized high-temperature spinel powders. Cubic Co_2MnO_4 (**HT-*c*-spinel)** and tetragonal $CoMn_2O_4$ (**HT-*t*-spinel)** were synthesized via a traditional sol-gel method (see Supplementary Method for preparation details) at relatively high temperatures[10].

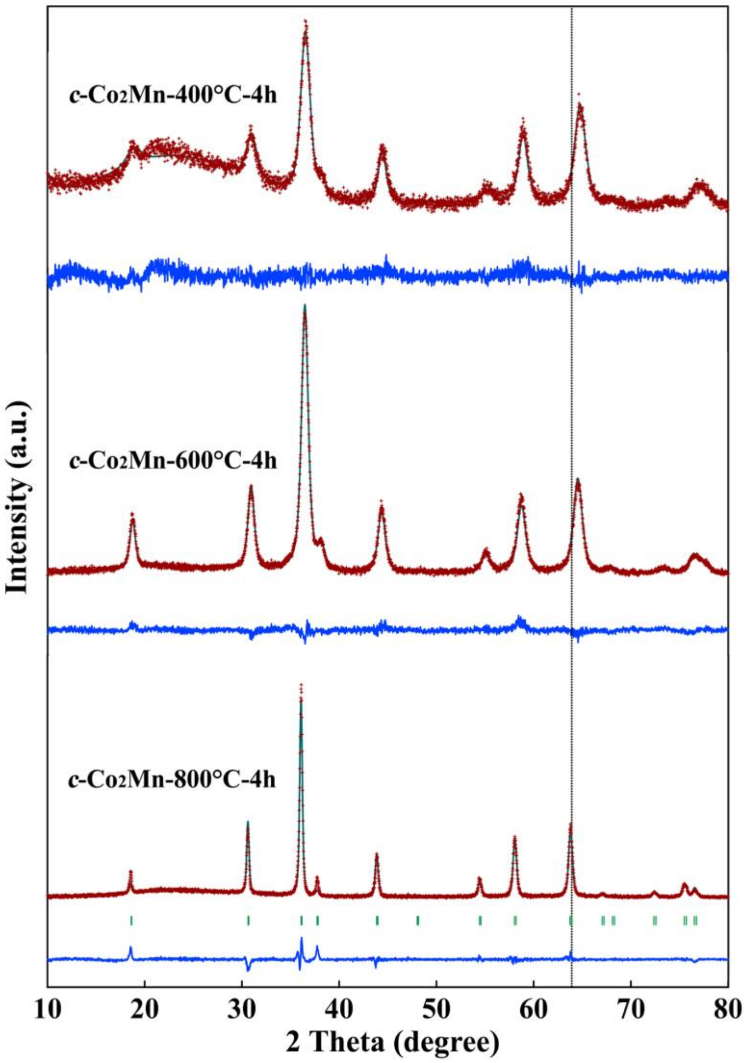

Supplementary Figure 13 | Rietveld refined X-ray patterns of *c*-Co_2Mn residues after thermal treatment at various temperatures. The sample was separately heated to 400, 600, and 800 °C in air and maintained at each temperature for 4 h. With increasing heating temperature, the diffraction peaks become sharper and shift to lower angles. From Rietveld refined data (Supplementary Table 7), it can be deduced that the cell parameter *a* and $(B\text{-}O)_{oct}$ distances of *c*-Co_2Mn progressively increase after thermal treatment at evaluated temperature. It can be explained by the reduction of Mn cations from Mn^{4+} to Mn^{3+} that has a longer ionic radius (0.530 versus 0.645 Å). This result has also been observed in cubic cobalt manganese spinel obtained at 400 and 600 °C[11].

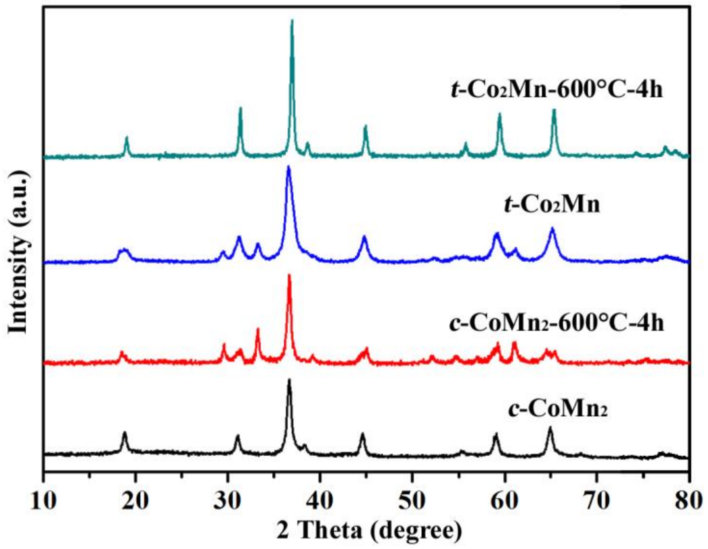

Supplementary Figure 14 | Comparison of X-ray diffraction curves of *c*-$CoMn_2$, *t*-Co_2Mn and corresponding residues after thermal treatment at 600 °C for 4 h in air. Evidently, *c*-$CoMn_2$ and *t*-Co_2Mn transform to tetragonal spinel (JCPDS card no.77-0471) and cubic phase (JCPDS card no.23-1237), respectively, after being heated at 600 °C for 4 h. As for $CoMn_2O_4$ and Co_2MnO_4, the thermodynamic stable phases are tetragonal and cubic structures. Thus, the unusual spinels (*c*-$CoMn_2$ and *t*-Co_2Mn) in our case are not maintained at high temperature and show structural transformation to generally thermodynamically stable phases. The tetragonal⇔cubic transition phenomenon has been observed at 900 °C in related systems[6]. This phase transformation may explain the successful controllable synthesis of phase and composition in our ambient experimental conditions, especially the *c*-$CoMn_2$ and *t*-Co_2Mn, which could not be achieved through traditional high-temperature route.

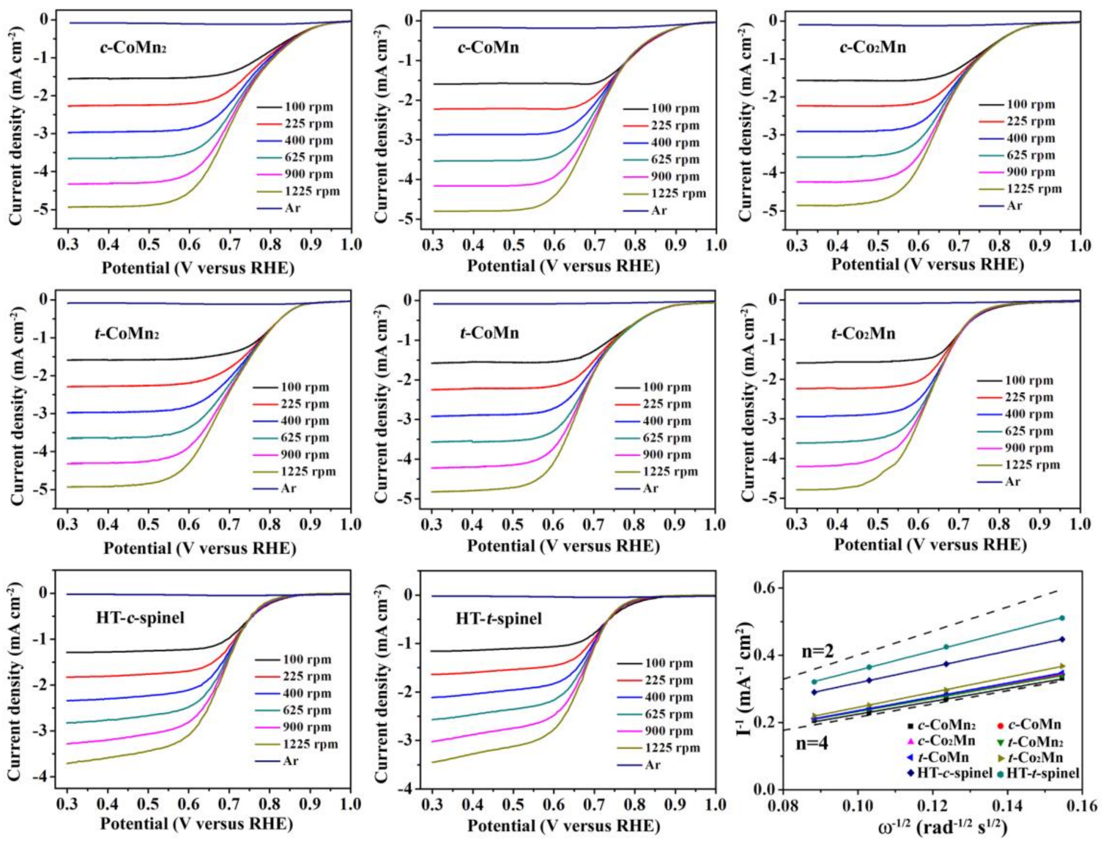

Supplementary Figure 15 | ORR electrocatalytic properties of CoMnO spinels. Rotation-speed-dependent current-potential curves of different catalyst-modified electrodes in O_2- or Ar-saturated 0.1 M KOH at 5 mV s^{-1} and the fitted Koutecky-Levich (K-L) curves at 0.5 V. The oxygen reduction current increases with rotation speed owing to efficient O_2 flow to the catalyst surface. In each panel, the polarization curve obtained in Ar is displayed for comparison, showing negligible current-voltage response. The obviously different currents detected in O_2 and Ar-saturated electrolyte indicate that the current-potential-responses are related to catalytic ORR. The voltammograms in O_2 are corrected by that in Ar to minimize possible effect of capacitive current. The constructed K-L curves are fitted well with linear relationship. The linear lines corresponding to two or four-electron (n = 2, 4) reduction were listed for comparison. Accordingly, CoMnO spinels synthesized under mild condition catalyze the ORR via a quasi-4e$^-$ pathway, which is desirable for promoting high-efficiency electrocatalytic ORR. Construction of K-L curves is described in Supplementary Note 5.

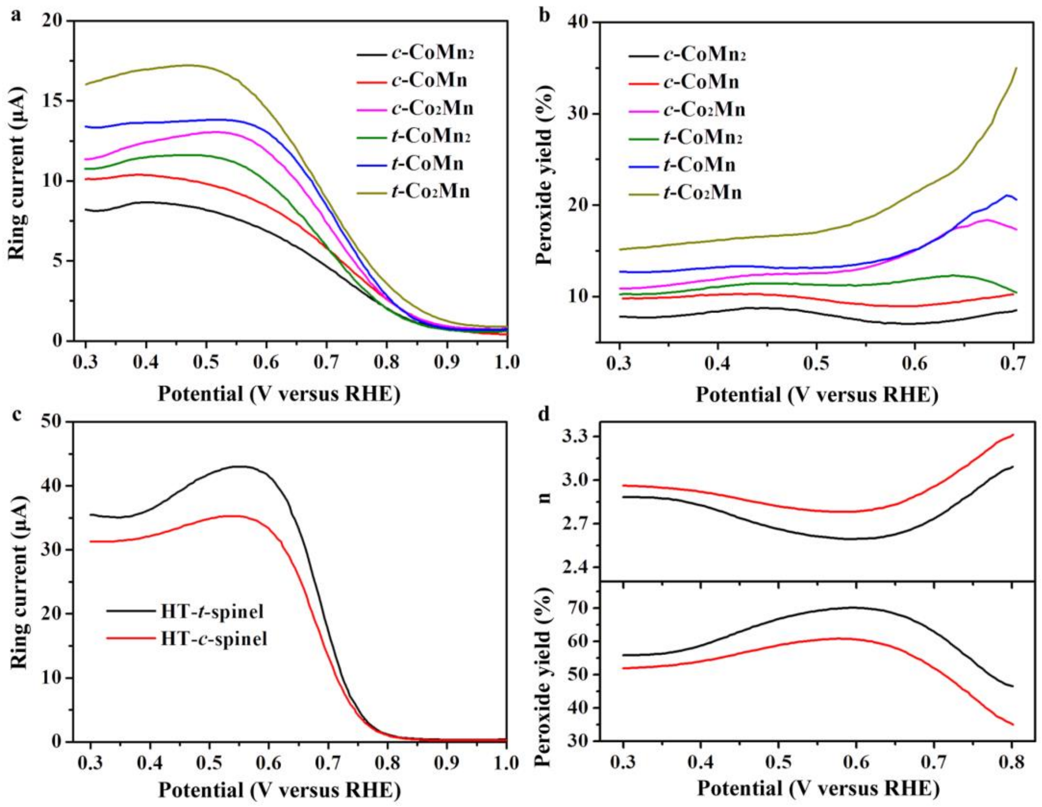

Supplementary Figure 16 | RRDE measurements. (**a**) Ring currents of synthesized nanocrystalline CoMnO at 900 rpm and (**b**) determined generated peroxide percentage with respect to the total oxygen reduction products. (**c**) Ring currents of high-temperature spinels at 900 rpm and (**d**) calculated electron transfer number (n) (top) and peroxide yield (bottom) at various potentials (see calculated details in Supplementary Note 4).

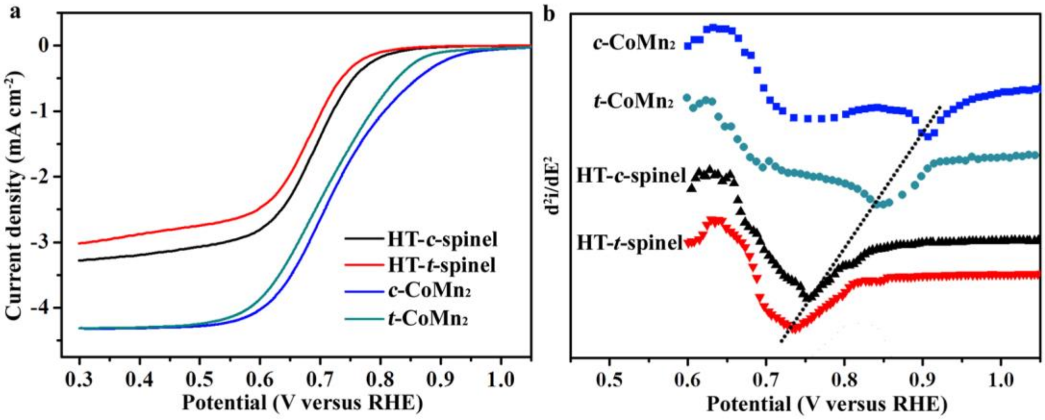

Supplementary Figure 17 | Comparison of ORR activity of CoMnO spinels synthesized under mild and high-temperature conditions. (**a**) Voltammograms of different catalysts modified glass carbon electrodes at 900 rpm in O_2-saturated 0.1 M KOH. (**b**) The quadratic differential curves constructed from data in **a**. The nanocrystalline *c*- and *t*-$CoMn_2$ are more active than the counterpart high-temperature spinels in terms of lower onset overpotential (also clearly seen from the peak values in **b**), higher half-wave potential and larger reduction current.

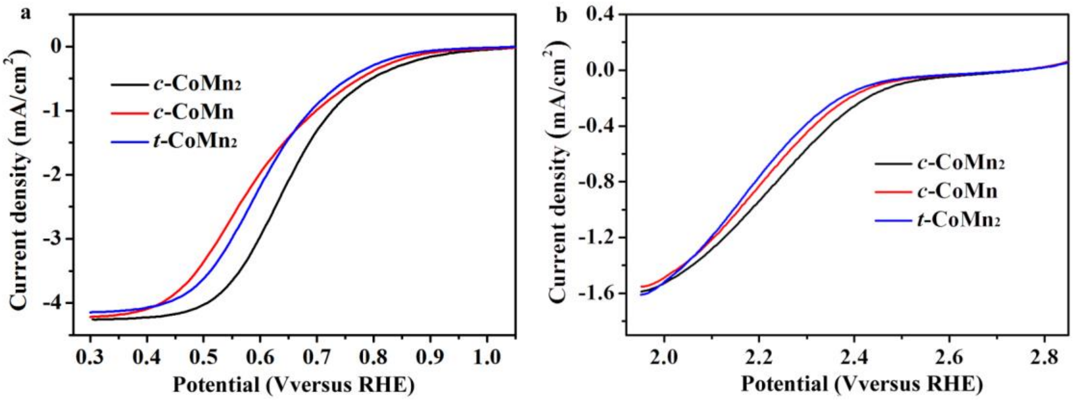

Supplementary Figure 18 | ORR activity in neutral and non-aqueous electrolyte. Linear sweeping voltammograms of selected spinels tested in O_2-saturated (**a**) 0.1 M KCl and (**b**) 1.0 M LiTFSI (lithium bis-(trifluoromethanesulfonyl)-imide) in TEGDME (tetraethylene glycol dimethyl ether) electrolyte at 900 rpm. Clearly, the nanocrystalline spinels exhibit considerable ORR activity in neutral or organic medium although the reduction onset potentials and half-wave potentials are slightly lower than those in alkaline solution.

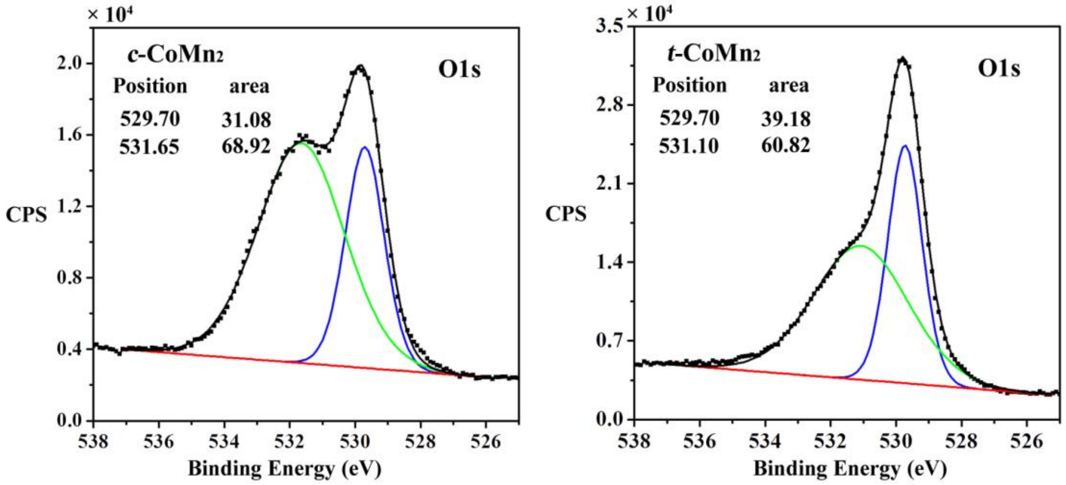

Supplementary Figure 19 | O1s XPS spectra of synthesized *c*-$CoMn_2$ and *t*-$CoMn_2$. The asymmetric O1s spectra can be deconvoluted into two peaks centered at ~529.7 and ~531.5 eV, which correspond to the lattice oxygen and surface adsorbed oxygen-containing species (such as hydroxide or water)[12,13], respectively. Particularly, the relative ratio of peak intensity and area of surface to lattice oxygen of cubic spinel are obviously higher than those of tetragonal spinel, indicating stronger oxygen binding ability of the cubic phase.

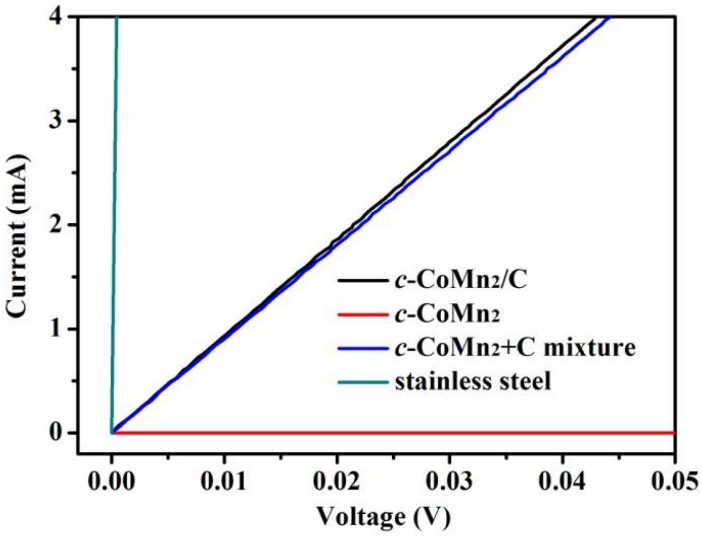

Supplementary Figure 20 | Electrical conductivity measurements. The electrical conductivities are determined to be 1.01, 5.4 × 10^3 and 1.3 × 10^4 S m^{-1} for *c*-$CoMn_2$, *c*-$CoMn_2$+C mixture and *c*-$CoMn_2$/C hybrid, respectively. The electrical conductivity of *c*-$CoMn_2$ is comparable with that reported in literature[14]. Notably, the electrical conductivity of *c*-$CoMn_2$ is around 3 orders of magnitude higher than that of binary spinel oxides such as Co_3O_4 and Mn_3O_4 and drastically higher than that of manganese oxides with nominal single valence state (e.g., MnO)[15–17]. Test procedures are described in Supplementary Method.

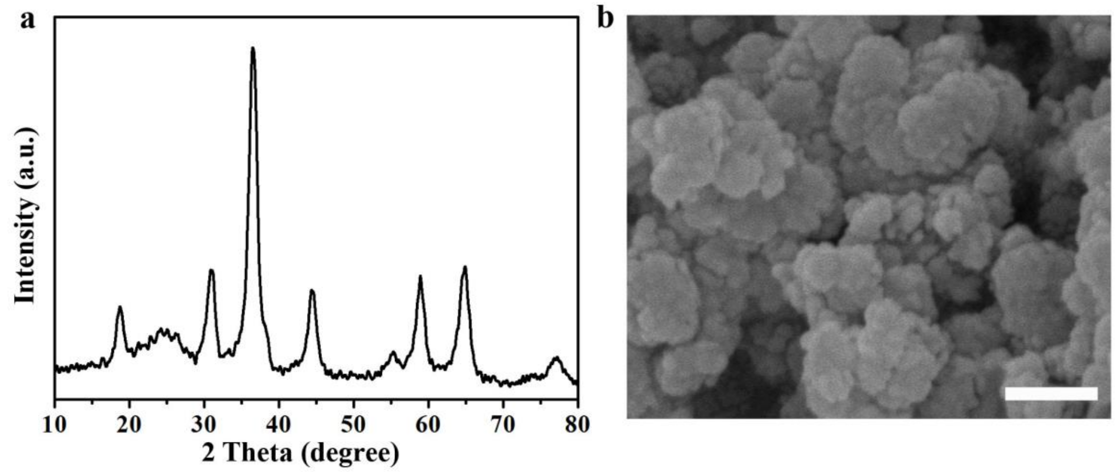

Supplementary Figure 21 | Characterization of *c*-$CoMn_2$/C hybrid. XRD (**a**) and SEM image (**b**) of the synthesized *c*-$CoMn_2$/C hybrid. Scale bar in (**b**) is 100 nm.

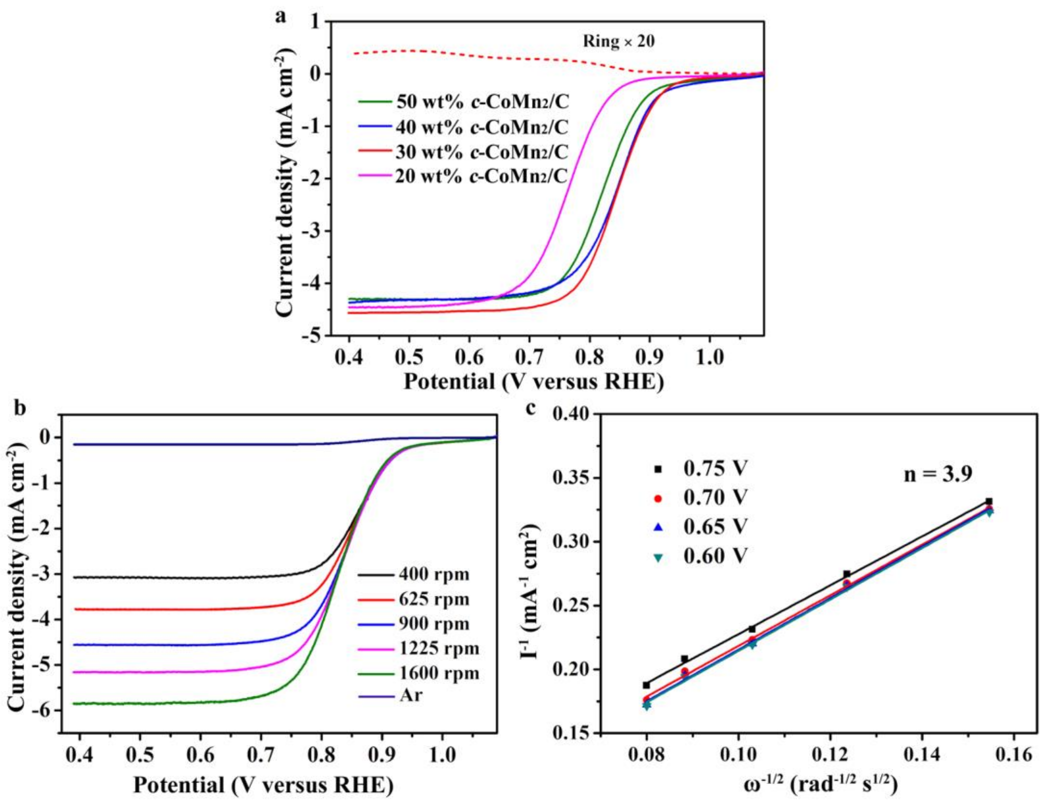

Supplementary Figure 22 | Catalytic activity of *c*-$CoMn_2$/C hybrid. (**a**) Influence of different proportion of conductive carbon on the ORR activity of as-prepared *c*-$CoMn_2$/C composite with rotation rate of 900 rpm. The carbon amount was optimized at ~70 wt.% based on the more positive oxygen reduction onset and half-wave potentials as well as the larger diffusion current density. The collected ring current of 30 wt.% *c*-$CoMn_2$/C hybrid is also shown (dotted line). (**b**) Rotating rate dependence of 30 wt.% *c*-$CoMn_2$/C catalyst in O_2- or Ar-saturated 0.1 M KOH at 5 mV s^{-1} and (**c**) Koutecky-Levich (K-L) curves constructed at different potentials.

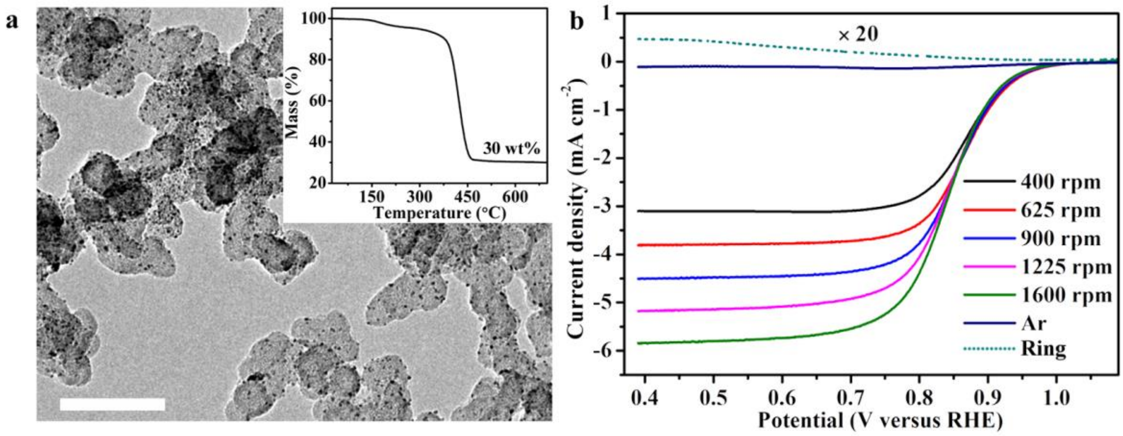

Supplementary Figure 23 | Characterization and ORR performance of Pt/C. (**a**) TEM image of prepared Pt/C (Scale bar 100 nm). Inset shows the thermogravimetry curve measured in air with Pt 30 wt.% mass loading. (**b**) Rotation-speed-dependent current-potential curves of Pt/C catalyst in O_2-or Ar-saturated 0.1 M KOH solution. The collected ring voltammetry at rotating rate of 900 rpm is also shown (dotted line).

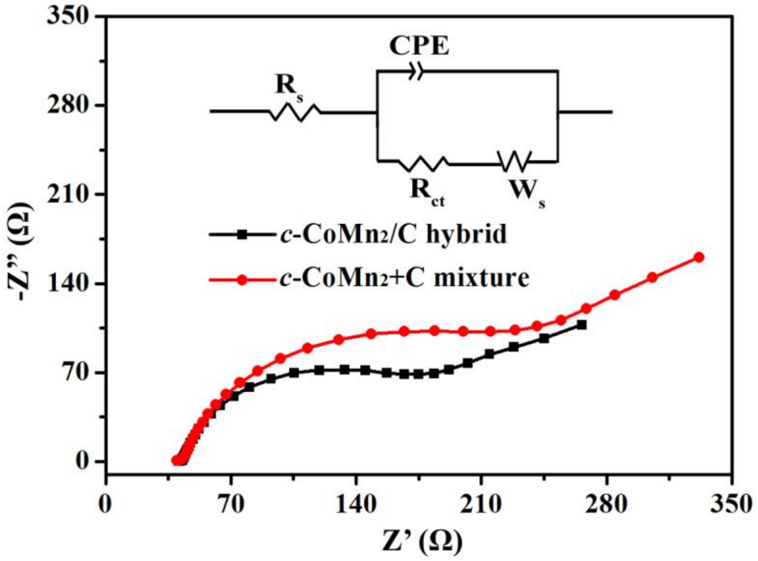

Supplementary Figure 24 | Impedance spectra. Electrochemical impedance spectra of *c*-$CoMn_2$/C hybrid and *c*-$CoMn_2$+C mixture at 0.7 V versus RHE within a three-electrode system. The equivalent circuit model (inset)[18] is employed to fit the electrochemical impedance of the oxygen electrocatalytic reduction reaction process. R_s is the sum of the electrode and electrolyte resistance, CPE is the double layer capacity, R_{ct} represents the charge transfer resistance and W_s stands for Warburg impedance within the diffusion component. In the Nyquist curves, a much smaller semicircle of *c*-$CoMn_2$/C hybrid is observed than that of *c*-$CoMn_2$+C mixture, indicating a more efficient charge transfer during the ORR process on *c*-$CoMn_2$/C hybrid.

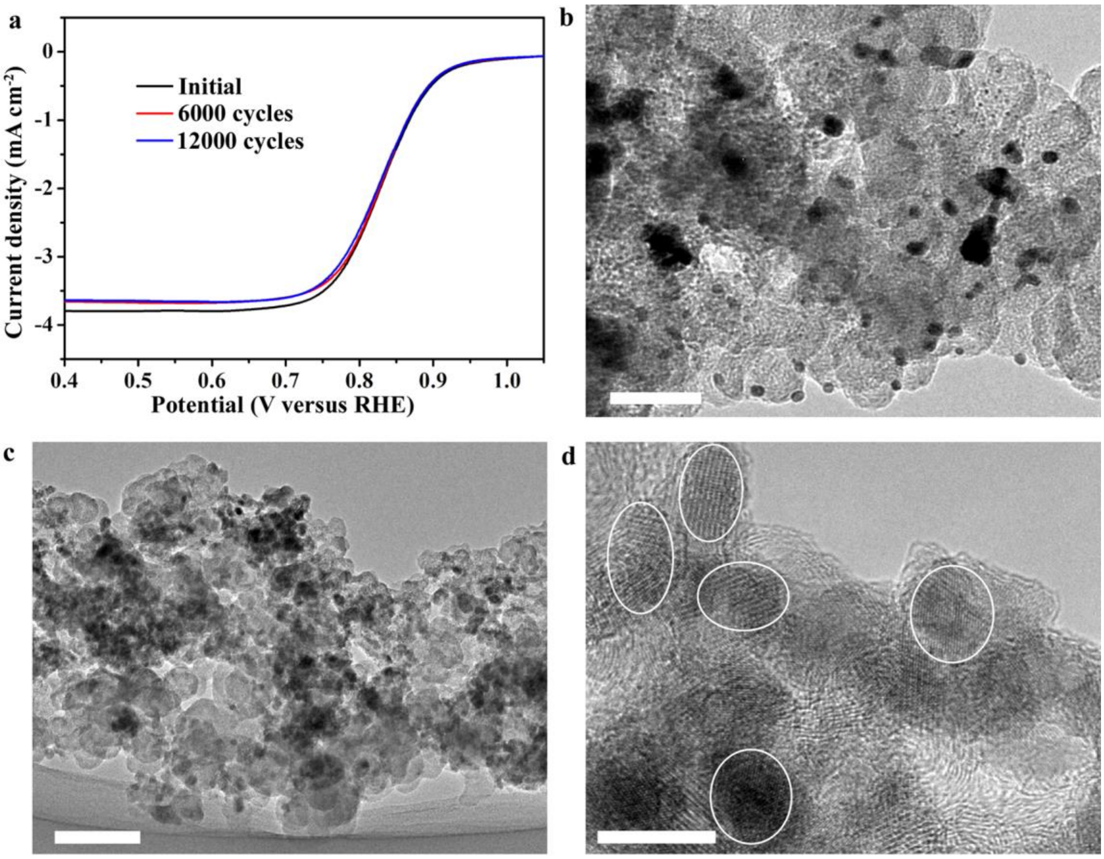

Supplementary Figure 25 | Stability measurements. (**a**) Durability assessment of *c*-$CoMn_2$/C hybrid with polarization curves at 625 rpm after 0, 6000 and 12000 cycles. Despite a little decreased current (~4.1%), there is no obvious difference between the polarization curves after 0, 6000 and 12000 cyclic voltammograms (CVs) cycles. (**b**) TEM characterization of Pt/C catalyst after 2000 repeated CV cycles and (**c**,**d**) *c*-$CoMn_2$/C hybrid after 12000 CV cycles. Circles in (**d**) mark some of spinel nanoparticles which are still tightly embedded in porous carbon matrix even after 12,000 repeated potential scanning processes. Scale bars, 100 nm (**b,c**) and 10 nm (**d**), respectively.

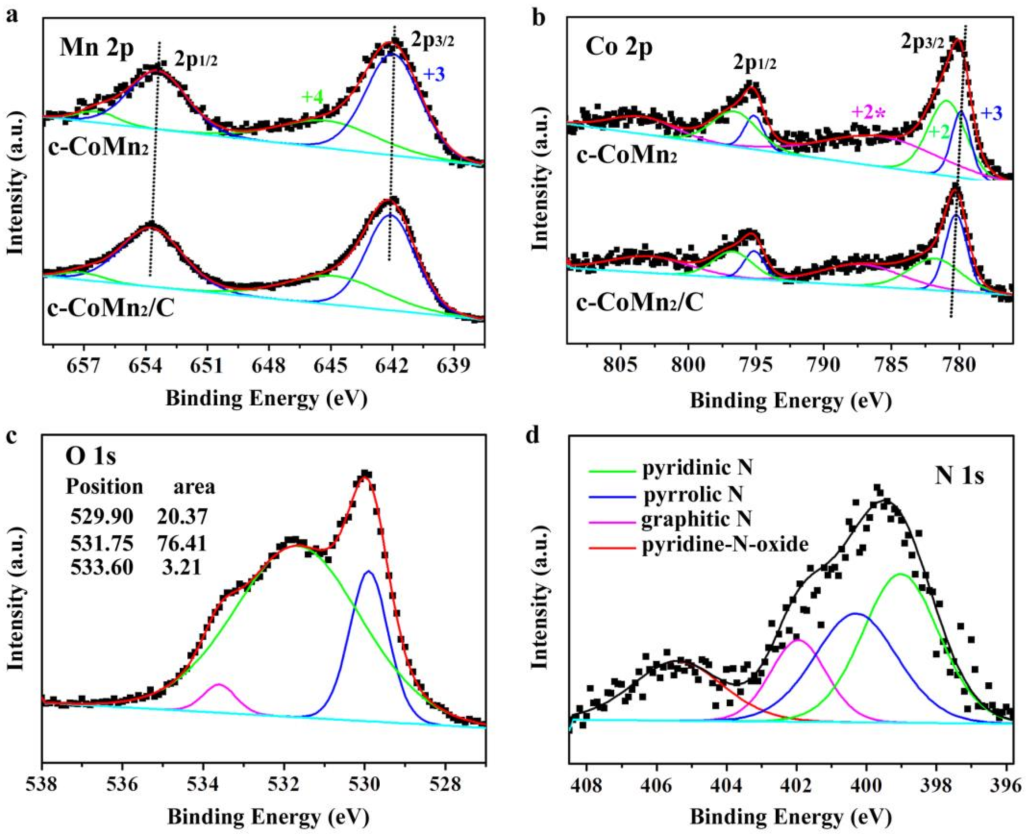

Supplementary Figure 26 | XPS characterization. High-resolution X-ray photoelectron spectroscopy (XPS) data of (**a**) Mn 2p, (**b**) Co 2p of *c*-$CoMn_2$ and *c*-$CoMn_2$/C, (**c**) O 1s and (**d**) N 1s of *c*-$CoMn_2$/C. The Mn 2p spectra (**a**) features two strong spin-orbit peaks of $2p_{3/2}$ centered at ~642.1 eV and $2p_{1/2}$ at ~653.5 eV. By refined fitting, the spectrum of the hybrid can be assigned to the coexistence of Mn (III) and Mn(IV), which is consistent with the results of neat *c*-$CoMn_2$. It should be noted that the positive shift (+0.12 eV) of the Mn binding energy in the hybrid is attributed to the covalent coupling effect between the *c*-$CoMn_2$ spinel and the carbon support[19]. Similarly, the Co 2p peaks (**b**) also confirm the mixed valence of +2 and +3 in the hybrid. The Co $2p_{3/2}$ and Co $2p_{1/2}$ spectra are accompanied by two obvious shake-up satellite peaks (785.9 and 803.4 eV), which again proves the presence of Co(II). The positive peak shift (+0.37 eV) is profound for Co cations. The stronger oxygen adsorption ability (**c**) for *c*-$CoMn_2$/C is also revealed by the higher ratio of peak intensity and area of surface oxygen species than that for pure *c*-$CoMn_2$ (76.41 % vs 68.92 %, see Supplementary Fig. 19). The total N content is 3.5 at% (**d**). The N 1s can be deconvoluted into four types centered at 399.0 eV (37.55 %), 400.3 eV (29.71 %), 401.9 eV (15.05 %), and 405.4 eV (17.69 %), which

correspond to pyridinic N, pyrrolic N, graphitic N and pyridine-N-oxide types, respectively. Pyridinic N and graphitic N are generally considered as active sites for the ORR[20]. Particularly, all the peaks shift positively to higher binding energy due to the covalent coupling effect between the *c*-$CoMn_2$ spinel and carbon support[19]. The peak shifts of N 1s reaches +0.8 eV, as a result of the strong interaction of C-N-transition metal. The obvious shift of peak position serves as an evidence for the strong covalent coupling between the spinel and carbon. This hybridization effect favors superior ORR activity and stability compared to that of physically mixed spinel-carbon counterpart.

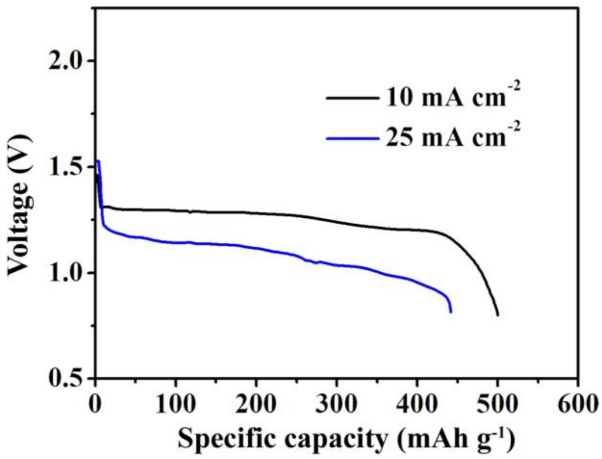

Supplementary Figure 27 | Performance of Zn-air batteries. Galvanostatic discharge curves of Zn-air batteries based on the prepared *c*-$CoMn_2$/C cathode catalyst at two different current densities. Specific capacity was calculated to the mass of the Zn anode. Accordingly, the specific capacity normalized to the mass of consumed Zn was ~500 mAh g^{-1} at 10 mA cm^{-2}, corresponding to a high energy density about 650 Wh kg^{-1}. The fabrication procedures of Zn-air battery are provided in Supplementary Method.

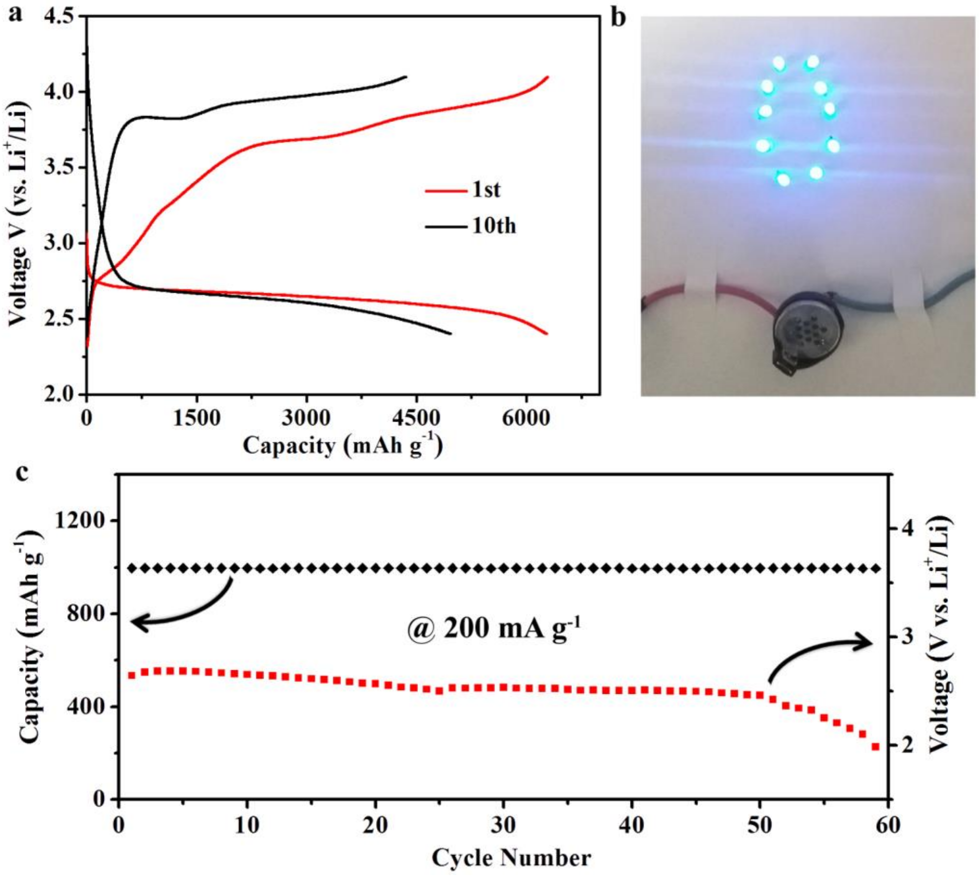

Supplementary Figure 28 | Performance of Li-air batteries. Li-air batteries based on the *c*-$CoMn_2$ spinel cathode electrocatalyst. (**a**) Discharge/charge curves at a current density of 200 mA g^{-1} within 2.4–4.1 V. (**b**) One assembled coin-type Li-air cell powers an "O" shape blue light that contains 10 LEDs. Under air atmosphere, the light was lit for more than three hours by a battery. (**c**) Voltage profiles and cycling performance at 200 mA g^{-1} with a limited capacity of 1000 mAh g^{-1}. The batteries could maintain ~60 cycles with discharge voltage above 2 V. The average energy density of the battery reaches 1560 Wh Kg^{-1} based on the total catalyst (2600 Wh Kg^{-1} based on carbon). The current density and specific capacity are normalized to the mass of carbon involved in the electrodes. Detailed procedures for Li-air battery assembly are described in Supplementary Method.

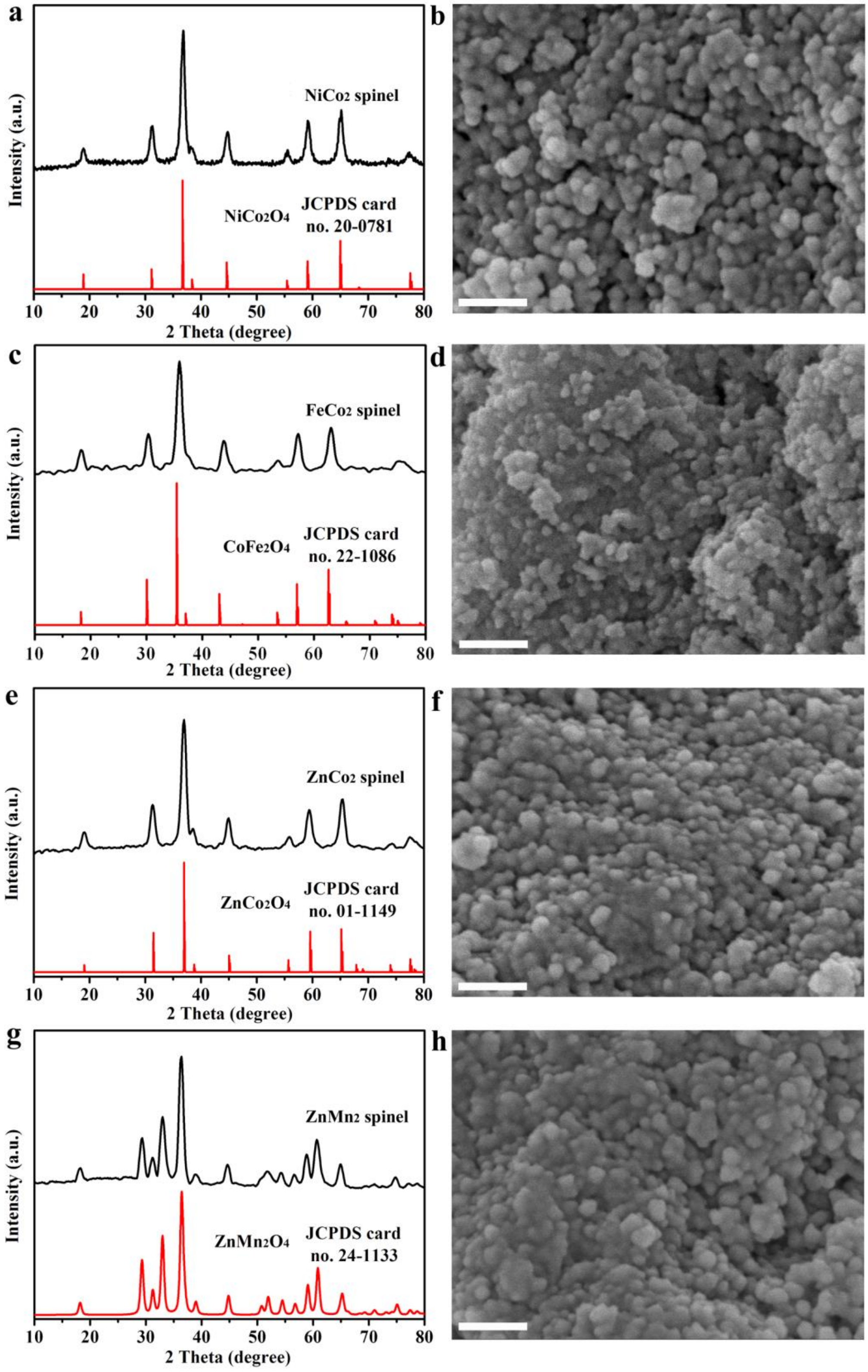

Supplementary Figure 29 | XRD and SEM of other prepared spinel oxides. (**a,c,e,g**) XRD patterns and (**b,d,f,h**) SEM images of (**a**,**b**) $NiCo_2O_4$, (**c**,**d**) $FeCo_2O_4$, (**e**,**f**) $ZnCo_2O_4$ and (**g**,**h**) $ZnMn_2O_4$ spinels. In (**a,c,e,g**), the corresponding standard XRD profiles with JCPDS card numbers are also listed for reference.

In (**b,d,f,h**), all scale bars are 100 nm. Synthesis details are described in Supplementary Method. XRD profiles of as-prepared samples match well with the corresponding standard patterns. Peak broadening implies small crystalline size. The SEM images show nanoscaled particulate shape. These results suggest that the present solution-based synthetic route could be applicable to a variety of spinel oxide nanomaterials.

Supplementary Table 1. Structural parameters of $(Co_{0.87}Y_{0.13})_{8a}(Mn_{1.76}Y_{0.24})_{16d}O_4$ detected in the synthesized *c*-$CoMn_2$. Related discussion is shown in Supplementary Note 1.

Atom	Site	g	x	y	z
Co	8a	0.87(3)	1/8	1/8	1/8
Mn	16d	0.88(4)	1/2	1/2	1/2
O	32e	1	0.2635(4)	0.2635(4)	0.2635(4)

Space group: Fd-3m (No. 227-2). Cell parameters: a = b = c = 8.182(7) Å. R_{wp} = 7.00 %, R_p = 5.48 %, R_I = 1.31 %, and S = 1.00. g: occupancy. x, y and z: atomic coordinates.

Supplementary Table 2. Structural parameters of $(Co_{0.92}Y_{0.08})_{8a}(Co_{0.46}Mn_{1.40}Y_{0.14})_{16d}O_4$ detected in the synthesized *c*-CoMn.

Atom	Site	g	x	y	z
Co1	8a	0.92(2)	1/8	1/8	1/8
Co2	16d	0.23(2)	1/2	1/2	1/2
Mn	16d	0.70(3)	1/2	1/2	1/2
O	32e	1	0.2653(2)	0.2653(2)	0.2653(2)

Space group: Fd-3m (No. 227-2). Cell parameters: a = b = c = 8.164(8) Å. R_{wp} = 6.89 %, R_p = 5.47 %, R_I = 1.60 %, and S = 1.05. g: occupancy. x, y and z: atomic coordinates.

Supplementary Table 3. Structural parameters of $(Co_{0.94}Y_{0.06})_{8a}(Co_{0.94}Mn_{0.92}Y_{0.14})_{16d}O_4$ detected in the synthesized *c*-Co_2Mn.

Atom	Site	g	x	y	z
Co1	8a	0.94(2)	1/8	1/8	1/8
Co2	16d	0.47(4)	1/2	1/2	1/2
Mn	16d	0.46(3)	1/2	1/2	1/2
O	32e	1	0.2586(4)	0.2586(4)	0.2586(4)

Space group: Fd-3m (No. 227-2). Cell parameters: a = b = c = 8.137(5) Å. R_{wp} = 9.40 %, R_p = 7.13 %, R_I = 4.69 %, and S = 1.45. g: occupancy. x, y and z: atomic coordinates.

Supplementary Table 4. Structural parameters of $(Co_{0.68}Mn_{0.32})_{4a}(Co_{0.36}Mn_{1.64})_{8d}O_{3.96}$ detected in the synthesized *t*-$CoMn_2$.

Atom	Site	g	x	y	z
Co1	4a	0.68(4)	0	0	0

Mn1	4a	0.32(1)	0	0	0
Co2	8d	0.18(5)	0	1/4	5/8
Mn2	8d	0.82(3)	0	1/4	5/8
O	16h	0.99(2)	0	0.2157(4)	0.3832(8)

Space group: I41/amd (No. 141). Cell parameters: a = b = 5.755(6) Å and c = 9.394(8) Å. R_{wp} = 11.38 %, R_p = 9.05 %, R_I = 4.27 %, and S = 2.08. g: occupancy. x, y and z: atomic coordinates.

Supplementary Table 5. Structural parameters of $(Co_{0.55}Mn_{0.45})_{4a}(Co_{0.90}Mn_{1.10})_{8d}O_{3.68}$ detected in the synthesized *t*-CoMn.

Atom	Site	g	x	y	z
Co1	4a	0.55(3)	0	0	0
Mn1	4a	0.45(3)	0	0	0
Co2	8d	0.45(4)	0	1/4	5/8
Mn2	8d	0.55(2)	0	1/4	5/8
O	16h	0.92(1)	0	0.2156(4)	0.3450(5)

Space group: I41/amd (No. 141). Cell parameters: a = b = 5.738(2) Å and c = 9.289(1) Å. R_{wp} = 11.70 %, R_p = 9.45 %, R_I = 4.97 %, and S = 1.69. g: occupancy. x, y and z: atomic coordinates.

Supplementary Table 6. Structural parameters of $(Co_{0.76}Mn_{0.24})_{4a}(Co_{1.26}Mn_{0.74})_{8d}O_{3.80}$ detected in the synthesized *t*-Co_2Mn.

Atom	Site	g	x	y	z
Co1	4a	0.76(3)	0	0	0
Mn1	4a	0.24(3)	0	0	0
Co2	8d	0.63(2)	0	1/4	5/8
Mn2	8d	0.37(4)	0	1/4	5/8
O	16h	0.95(2)	0	0.2447(6)	0.4482(8)

Space group: I41/amd (No. 141). Cell parameters: a = b = 5.737(8) Å and c = 9.209(4) Å; R_{wp} = 11.16 %, R_p = 8.71 %, R_I = 4.16 %, and S = 1.71. g: occupancy. x, y and z: atomic coordinates.

Supplementary Table 7 | Comparison of cell parameters and Mn-O bond distances for *c*-Co_2Mn spinels after annealing at elevated temperature.

Sample	Cell parameter (Å)	$d_{(Co/Mn-O)oct}$[a] (Å)	R_{wp} (%)	R_p (%)	R_I (%)	S
c-Co_2Mn	8.137(5)	1.962	9.40	7.13	4.69	1.45
c-Co_2Mn-400°C-4h	8.140(5)	1.972	8.14	6.39	3.65	0.99
c-Co_2Mn-600°C-4h	8.212(3)	1.981	6.28	4.91	1.67	0.99
c-Co_2Mn-800°C-4h	8.265(5)	1.988	9.12	6.59	5.97	1.35

[a]Average distances of B-O bonds (B represents Co and/or Mn at the octahedral sites) in *c*-Co_2Mn spinel.

Supplementary Table 8 | Summary of Mn content/valence and electrochemical properties of CoMnO spinels and the benchmark Pt/C[a].

Sample	Mn content (%)	Mn valence	E_{onset} (V)	E_{half} (V)	I_k (mA cm^{-2})	I_{sa} (mA cm^{-2})	I_m (mA mg^{-1})	n	TOF (S^{-1})
c-$CoMn_2$	47.8	3.49	0.91	0.73	1.40	0.31	26.0	3.83	0.07
c-CoMn	35.4	3.45	0.89	0.71	0.98	0.16	18.2	3.80	0.05
c-Co_2Mn	23.2	3.28	0.85	0.67	0.60	0.10	11.15	3.75	0.03
t-$CoMn_2$	46.5	2.84	0.86	0.71	0.90	0.17	16.73	3.77	0.05
t-CoMn	35.1	2.76	0.87	0.68	0.67	0.15	12.45	3.74	0.03
t-Co_2Mn	22.9	2.67	0.78	0.64	0.15	0.04	2.79	3.66	0.008
c-$CoMn_2$/C			0.95	0.85	20.4		379.1	3.91	1.07
HT-*c*-spinel			0.80	0.69	0.18		3.35	2.82	
HT-*t*-spinel			0.77	0.67	0.10		1.86	2.66	
Pt/C			0.96	0.85	21.7		403.3	3.86	0.69

[a]E_{onset}, E_{half}, I_k, I_{sa}, I_m and n are onset potential, half-wave potential, kinetic current density based on geometric area of electrode, kinetic current density based on BET surface area, kinetic current density based on the mass of oxide, electron transfer number, respectively. I_k, I_{sa}, I_m, and TOF are determined

at 0.8 V and n is calculated at 0.5 V. TOF values are calculated by assuming all metal atoms are involved in the catalysis (details are given in Supplementary Note 6). All the data is based on the voltammograms at 900 rpm.

Supplementary Table 9 | ORR activities of comparative nonprecious electrocatalysts[a].

Catalyst	E_{onset} (V)	E_{half} (V)	I_s (mA cm^{-2})	I_k (mA cm^{-2})	I_m (mA mg^{-1})	n	Electrolyte	Reference
c-$CoMn_2$/C	0.95	0.83	5.7	3.58	66.3	3.91	0.1 M KOH	this work
$LaCu_{0.5}Mn_{0.5}O_3$	~0.80	~0.64	5.7				0.1 M KOH	5
$MnCo_2O_4$/ N-rmGO	0.95	0.88	~3.8	~9.9	~123.8	~3.85	1.0 M KOH	21
LT-$Li_{0.5}CoO_2$	~0.85	~0.68	~5.0				0.1 M KOH	22
Fe_3O_4/N-GAs	0.77	~0.56	~4.5			~3.8	0.1 M KOH	23
Nanostructured Mn oxide	~0.86	~0.75	~5.0	~0.56	~20.0		0.1 M KOH	24
Co_3O_4/N-rmGO	0.88	0.83	~5.0	~2.14	~30.6	3.9	0.1 M KOH	25
$CaMnO_3$ microsphere	0.96	0.76	5.7	1.39	15.3	3.96	0.1 M KOH	26
N, S, O-doped mesoporous carbon	0.85	~0.75	~4.0			3.5	0.1 M KOH	27

[a]I_s is saturated current density based on geometric area of electrode. I_k and I_m are determined at 0.85 V whereas n is calculated at 0.5 V versus RHE. The rotation rate is 1600 rpm.

Supplementary Note 1. Rietveld refinement of synthesized spinels.

In Table 1-7, the often-used R-values are listed to assess the fitting. $R_{wp,}$ R_p, R_I, and S represent R-weighted pattern, R-pattern, R-Bragg factor and 'goodness of fit' indicator, respectively. Occupation numbers were calculated by restricting the total Co/Mn ratio (determined from elemental analysis) and charge balance is achieved by formation of vacancies (Y) on the metal or O sites[28]. Numbers in parenthesis of Table 1-7 are the refinement standard deviations.

Supplementary Note 2. Mass-averaged particle size calculation based on the statistical data of TEM images.

The mass-averaged particle size (d_m) is determined based on equation 1 (Ref. 29):

$$d_m = \sqrt[3]{\frac{\sum_i n_i D_i^3}{N_T}} \tag{1}$$

where n_i is the number of particles of diameter D_i, N_T is the total number of sampled particles. For each sample, 80–110 particles are measured and counted.

Supplementary Note 3. The calculation of free Gibbs energy change.

The formation of Mn_7O_{13} from MnOOH is thermodynamically favorable based on the negative change of free Gibbs energy according to following reaction[30]:

$$7MnOOH + 5Co(NH_3)_6^{3+} + 5OH^- \rightarrow Mn_7O_{13} + 5Co(NH_3)_6^{2+} + 6H_2O$$

$$\Delta G^0 = \Delta_f G^0(Mn_7O_{13}) + 5\Delta_f G^0(Co(NH_3)_6^{2+}) + 6\Delta_f G^0(H_2O) - 7\Delta_f G^0(MnOOH) - 5\Delta_f G^0(Co(NH_3)_6^{3+}) - 5\Delta_f G^0(OH^-)$$

$$= -3207.2 + 5 \times (-157.3) + 6 \times (-234.54) - 7 \times (-503.93) - 5 \times (-189.5) - 5 \times (-157.28)$$

$$= -139.53 \text{ kJ mol}^{-1}.$$

Supplementary Note 4. Calculation of electron transfer number and percentage of peroxide.

The electron transfer number and percentage of peroxide species (relative to the total products) were calculated by equations 2 and 3 (Ref. 21, 31):

$$n = \frac{4NI_d}{NI_d + I_r} \tag{2}$$

$$HO_2^-(\%) = \frac{200I_r}{NI_d + I_r} \tag{3}$$

where I_d, I_r and N are the disk current, ring current and current collection efficiency of RRDE.

Supplementary Note 5. Construction of K-L plots.

The relationship between the measured currents (I) with various rotating speeds (ω) under fixed potentials can be expressed on the basis of the Koutechky-Levich (K-L) equation 4 (Ref. 21):

$$\frac{1}{I} = \frac{1}{I_k} + \frac{1}{I_d} = \frac{1}{I_k} + \frac{1}{B\omega^{1/2}} \tag{4}$$

$$I_k = nFkC_0;\ B = 0.62nFC_0D_{O_2}^{2/3}v^{-1/6}$$

where I, I_k and I_d are the measured, kinetic, and diffusion-limiting current densities, respectively. ω is the angular velocity in terms of rad s^{-1}, n is the transferred electron number during ORR process, F is the Faraday constant, k is the rate constant for electron transfer, C_0 is the O_2-saturated concentration in the electrolyte, D_{O_2} is the O_2 diffusion coefficient, and ν is the kinetic viscosity of the electrolyte. The kinetic current is obtained from the mass-diffusion correction based on equation 5:

$$I_k = \frac{I \times I_d}{I_d - I} \tag{5}$$

where I_k, I and I_d are the kinetic, measured and diffusion-limiting current densities, respectively.

Supplementary Note 6. Calculation of turnover frequency (*TOF*).

The *TOF* value is determined from equations 6 and 7 (Ref. 32):

$$TOF = \frac{I_k \times A}{4 \times e \times N_s} \tag{6}$$

$$N_s = ND = 6N\frac{v}{s}\frac{\sum_i n_i d_i^2}{\sum_i n_i d_i^3} \tag{7}$$

where I_k is the measured kinetic current density at overpotential of 430 mV and is determined according to equation 5, *A* is the electrode area, N_s and *N* is the number of active surface and total metal atoms on the electrode, *D* is the surface average dispersion (ratio of atoms on the surface to the total number of atoms in the particles), *v* and *s* represent the average volume and area per metal atom in unit cell respectively, and n_i is the frequency of appearance of particle of size d_i. The calculated *D* values are around 7.2%, 8.3%, 8.7%, 7.5%, 6.4% and 6.7% for *c*-$CoMn_2$, *c*-CoMn, *c*-Co_2Mn, *t*-$CoMn_2$, *t*-CoMn and *t*-Co_2Mn, respectively. The obtained *TOF* values are rough estimate and represent the lower limit, because some of these metal sites are electrochemically unaccessible. The calculated ORR *TOF* values in this case are comparable with previous reported ORR electrocatalysts[32].

Supplementary Methods:

Synthesis of CoMnO spinels using sol-gel route at high temperature. In a typical synthesis, 3 mmol total amount of $Co(CH_3COO)_2 \cdot 4H_2O$ and $Mn(CH_3COO)_2 \cdot 4H_2O$ (Co:Mn molar ratio 2:1 for cubic Co_2MnO_4 and 1:2 for tetragonal $CoMn_2O_4$) were dissolved in 10 mL deionized water. After adding 6.4 mmol citric acid, the solution was neutralized by 1.2 mL 25 wt.% ammonia water. Under magnetic stirring, 100 mL absolute ethanol was poured into the mixed solution. The water and ethanol liquid were evaporated in an airing oven under 100 ^oC to get the gel-like precursor. The final spinel products were obtained by heating the precursors at 600 ^oC for 18 h in air atmosphere. The peaks of prepared samples correspondingly match well with the tetragonal spinel (JCPDS card no. 77-0471) and the cubic spinel (JCPDS card no. 23-1237).

Electrical conductivity measurements. Around 200 mg of sample powders was pressed by 20 MPa into a 1.3 cm diameter, 0.05 cm thick chip. To measure the electrical resistivity, two stainless steel electrodes (1.6 cm diameter, 0.2 cm thick) were placed onto two faces of the chip and held by an alligator clip. The resistivity test was performed using Ametek Parstat 4000. A stainless steel chip (1.6 cm diameter, 0.2 cm thick, resistivity 6.9×10^{-7} $\Omega\cdot m$) was used as reference. The resistance values (R) of the samples were determined by voltmeter-ammeter method. The conductivity κ was deduced using relation: $\kappa = 1/\rho = h/(R \times S)$, where ρ is resistivity and S and h are the surface and thickness of the chips).

Zn-air battery assembly. The zinc-air battery was assembled by the zinc plate anode, porous air cathode and 25-30 mL aqueous electrolyte consisting of 6.0 M KOH without the use of separator. The air cathode was prepared by casting homogenous ink of *c*-$CoMn_2$/C (10 mg catalyst and 35 μL Nafion solution (5 wt.%, Sigma-Aldrich) in 1 mL mixed solvent of water/isopropyl alcohol (volume ratio: 3:1) on a 0.8 cm^2 carbon paper (Fuel Cell Store) and dried at 100 °C for 3 h to obtain a catalyst loading of ~2.0 mg/cm^2. Battery testing and cycling were performed at room temperature using LAND-CT2001A testing system.

Li-air battery assembly. The CoMnO spinel (30 wt.%) was mixed with conductive carbon additive (Vulcan XC-72, 60 wt.%) and polyvinylidene fluoride (PVdF, 10 wt.%). The homogenous ink was casted onto Ni foam current collector with a total mass loading of ~0.5 mg cm^{-2}. The battery was

assembled with lithium anode, glass fibre separator, and electrolyte of 1.0 M LiTFSI (lithium bis(trifluoromethane) sulfonamide) in TEGDME (tetraethylene glycol dimethyl ether).

Synthesis of other spinels. Four representative Co and Mn-based spinel oxides have been successfully synthesized using the similar oxidation-precipitation and crystallization route described in this study. Typically, to prepare cobalt-based spinel oxides ($NiCo_2O_4$, $FeCo_2O_4$ and $ZnCo_2O_4$), 4 mL aqueous ammonia (25 wt.%) was firstly dripped into 10 mL 0.2 M $Co(NO_3)_2$ solution under constant stirring at room temperature. After that, 5 mL of 0.2 M nickel/iron/zinc nitrate solution was added to the mixture, which was stirred for 120 min. Then, the mixture was evaporated and heated at 180 °C for 40 min, yielding nickel/iron/zinc cobalt spinel oxides. The preparation of $ZnMn_2O_4$ followed similar procedures by replacing $Co(NO_3)_2$ with $Mn(NO_3)_2$.

Supplementary References

1 Grush, M. M. *et al*. Correlating electronic structure with cycling performance of substituted $LiMn_2O_4$ electrode materials: a study using the techniques of soft X-ray absorption and emission. *Chem. Mater.* **12**, 659–664, (2000).

2 Yoon, W. S. *et al*. Oxygen contribution on Li-ion intercalation-deintercalation in $LiAl_yCo_{1-y}O_2$ investigated by O K-edge and Co L-edge X-ray absorption spectroscopy. *J. Electrochem. Soc.* **149**, A1305–A1309, (2002).

3 Yoon, W. S. *et al*. Investigation of the charge compensation mechanism on the electrochemically Li-ion deintercalated $Li_{1-x}Co_{1/3}Ni_{1/3}Mn_{1/3}O_2$ electrode system by combination of soft and hard X-ray absorption spectroscopy. *J. Am. Chem. Soc.* **127**, 17479–17487, (2005).

4 Kobayashi, S., Usui, T., Ikuta, H., Uchimoto, Y. & Wakihara, M. Soft X-ray absorption near edge structure analysis on lithium manganese oxide prepared by microwave heating. *J. Am. Ceram. Soc.* **87**, 1002–1007, (2004).

5 Suntivich, J. *et al.* Design principles for oxygen reduction activity on perovskite oxide catalysts for fuel cells and metal air batteries. *Nat. Chem.* **3**, 546–550 (2011).

6 Vila, E., Rojas, R. M., Martín de Vidales, J. L. & García-Martínez, O. Structural and thermal properties of the tetragonal cobalt manganese spinels $Mn_xCo_{3-x}O_4$ ($1.4 < x < 2.0$). *Chem. Mater.* **8**, 1078–1083 (1996).

7 Rojas, R. M., Vila, E., Garcia, O. & de Vidales, J. L. M. Thermal behaviour and reactivity of manganese cobaltites $Mn_xCo_{3-x}O_4$ ($0.0 \leq x \leq 1.0$) obtained at low temperature. *J. Mater. Chem.* **4**, 1635–1639 (1994).

8 Li, Y. G., Tan, B. & Wu, Y. Y. Freestanding mesoporous quasi-single-crystalline Co_3O_4 nanowire arrays. *J. Am. Chem. Soc.* **128**, 14258–14259 (2006).

9 Cheng, F. Y. *et al.* Rapid room-temperature synthesis of nanocrystalline spinels as oxygen reduction and evolution electrocatalysts. *Nat. Chem.* **3**, 79–84 (2011).

10 Lavela, P., Tirado, J. L. & Vidal-Abarca, C. Sol-gel preparation of cobalt manganese mixed oxides for their use as electrode materials in lithium cells. *Electrochim. Acta* **52**, 7986–7995 (2007).

11 Mateos, J. M. J., Morales, J. & Tirado, J. L. Cation-deficient Mn, Co spinel oxides obtained by

thermal decomposition of carbonate precursors. *J. Solid State Chem.* **82**, 87–94 (1989).

12 Tan, B. J., Klabunde, K. J. & Sherwood, M. A. XPS studies of solvated metal atom dispersed catalysts. Evidence for layered cobalt-manganese particles on alumina and silica. *J. Am. Chem. Soc.* **113**, 855–861 (1991).

13 Zhang, H. T. & Chen, X. H. Size-dependent X-ray photoelectron spectroscopy and complex magnetic properties of $CoMn_2O_4$ spinel nanocrystals. *Nanotechnology* **17**, 1384–1390 (2006).

14 Rousset, A. *et al.* Electrical properties of $Mn_{3-x}Co_xO_4$ ($0 \leq x \leq 3$) ceramics: An interesting system for negative temperature coefficient thermistors. *Int. J. Appl. Ceram. Technol.* **10**, 175–185 (2013).

15 Tareen, J. A. K. et al. Growth and electrical properties of pure and Ni-doped Co_3O_4 single crystals. *Mater. Res. Bull.* **19**, 989–997 (1984).

16 Bose, V. C. & Biju, V. Optical, electrical and magnetic properties of nanostructured Mn_3O_4 synthesized through a facile chemical route. *Physica E* **66**, 24–32 (2015).

17 Bhide, V. G. & Dani, R. H. Electrical conductivity in oxides of manganese and related compounds. *Physica* **27**, 21–826 (1961).

18 Liu, H. *et al.* Highly graphitic carbon black-supported platinum nanoparticle catalyst and its enhanced electrocatalytic activity for the oxygen reduction reaction in acidic medium. *Electrochim. Acta* **93**, 25–31 (2013).

19 Ge, X. *et al.* Dual-phase spinel $MnCo_2O_4$ and spinel $MnCo_2O_4$/nanocarbon hybrids for electrocatalytic oxygen reduction and evolution. *ACS Appl. Mater. Interfaces* **6**, 12684–12691 (2014).

20 Zhang, Y. *et al.* Engineering self-assembled N-doped graphene-carbon nanotube composites towards efficient oxygen reduction electrocatalysts. *Phys. Chem. Chem. Phys.* **16**, 13605–13609 (2014).

21 Liang, Y. Y. *et al.* Covalent hybrid of spinel manganese–cobalt oxide and graphene as advanced oxygen reduction electrocatalysts. *J. Am. Chem. Soc.* **134**, 3517–3523 (2012).

22 Maiyalagan, T. *et al.* Spinel-type lithium cobalt oxide as a bifunctional electrocatalyst for the oxygen evolution and oxygen reduction reactions. *Nat. Commun.* **5**, 3949 (2014).

23 Wu, Z.-S. *et al.* 3D Nitrogen-doped graphene aerogel-supported Fe_3O_4 nanoparticles as efficient

electrocatalysts for the oxygen reduction reaction. *J. Am. Chem. Soc.* **134**, 9082–9085 (2012).

24 Gorlin, Y. & Jaramillo, T. F. A bifunctional nonprecious metal catalyst for oxygen reduction and water oxidation. *J. Am. Chem. Soc.* **132**, 13612–13614 (2010).

25 Liang, Y. Y. *et al.* Co_3O_4 nanocrystals on graphene as a synergistic catalyst for oxygen reduction reaction. *Nat. Mater.* **10**, 780-786 (2011).

26 Han, X. P. *et al.* Porous calcium-manganese oxide microspheres for electrocatalytic oxygen reduction with high activity. *Chem. Sci.* **4**, 368-376 (2013).

27 Cheon, J. Y. *et al.* Intrinsic relationship between enhanced oxygen reduction reaction activity and nanoscale work function of doped carbons. *J. Am. Chem. Soc.* **136**, 8875–8878 (2014).

28 Srdić, V. V., Rakić, S. & Cvejić, Ž. Aluminum doped zirconia nanopowders: Wet-chemical synthesis and structural analysis by Rietveld refinement. *Mater. Res. Bull.* **43**, 2727–2735 (2008).

29 Sudsakorn, K. & Turton, R. Nonuniformity of particle coating on a size distribution of particles in a fluidized bed coater. *Powder Technol.* **110**, 37–43 (2000).

30 Bard, A. J., Parsons, R. & Jordan, J. *Standard potentials in aqueous solution* (Marcel Dekker, New York, 1985).

31 Bard, A.J. & Faulkner, L. R., *Electrochemical Methods: Fundamentals and Applications*, (Wiley, New York, 2000).

32 Paulus, U. A. *et al.* Oxygen Reduction on Carbon-Supported Pt−Ni and Pt−Co Alloy Catalysts. *J. Phys. Chem. B* **106**, 4181–4191 (2002).
